# SPT6 recruits SND1 to co‐activate human telomerase reverse transcriptase to promote colon cancer progression

**DOI:** 10.1002/1878-0261.12878

**Published:** 2021-01-12

**Authors:** Chaoliang Diao, Ping Guo, Wenjing Yang, Yao Sun, Yina Liao, Yue Yan, Anshi Zhao, Xin Cai, Jiaojiao Hao, Sheng Hu, Wendan Yu, Manyu Chen, Ruozhu Wang, Wenyang Li, Yan Zuo, Jinjin Pan, Chunyu Hua, Xiaona Lu, Wenhua Fan, Zongheng Zheng, Wuguo Deng, Guangyu Luo, Wei Guo

**Affiliations:** ^1^ Institute of Cancer Stem Cells and the First Affiliated Hospital Dalian Medical University China; ^2^ State Key Laboratory of Oncology in South China Collaborative Innovation Center of Cancer Medicine Sun Yat‐sen University Cancer Center Guangzhou China; ^3^ The Third Affiliated Hospital Sun Yat‐sen University Guangzhou China

**Keywords:** colorectal cancer, hTERT, SND1, SPT6, transcription regulation

## Abstract

Human telomerase reverse transcriptase (hTERT) plays an extremely important role in cancer initiation and development, including colorectal cancer (CRC). However, the precise upstream regulatory mechanisms of hTERT in different cancer types remain poorly understood. Here, we uncovered the candidate transcriptional factor of hTERT in CRC and explored its role and the corresponding molecular mechanisms in regulating hTERT expression and CRC survival with an aim of developing mechanism‐based combinational targeting therapy. The possible binding proteins at the hTERT promoter were uncovered using pull‐down/mass spectrometry analysis. The regulation of SPT6 on hTERT expression and CRC survival was evaluated in human CRC cell lines and mouse models. Mechanistic studies focusing on the synergy between SPT6 and staphylococcal nuclease and Tudor domain containing 1 (SND1) in controlling hTERT expression and CRC progression were conducted also in the above two levels. The expression correlation and clinical significance of SPT6, SND1, and hTERT were investigated in tumor tissues from murine models and patients with CRC in situ. SPT6 was identified as a possible transcriptional factor to bind to the hTERT promoter. SPT6 knockdown decreased the activity of hTERT promoter, downregulated the protein expression level of hTERT, suppressed proliferation, invasion, and stem‐like properties, promoted apoptosis induction, and enhanced chemotherapeutic drug sensitivity *in vitro*. SPT6 silencing also led to the delay of tumor growth and metastasis in mice carrying xenografts of human‐derived colon cancer cells. Mechanistically, SND1 interacted with SPT6 to co‐control hTERT expression and CRC cell proliferation, stemness, and growth *in vitro* and *in vivo*. SPT6, SND1, and hTERT were highly expressed simultaneously in CRC tissues, both from the murine model and patients with CRC in situ, and pairwise expression among these three factors showed a significant positive correlation. In brief, our research demonstrated that SPT6 synergized with SND1 to promote CRC development by targeting hTERT and put forward that inhibiting the SPT6‐SND1‐hTERT axis may create a therapeutic vulnerability in CRC.

## Graphical abstract

Colorectal cancer (CRC) is continuously rising among young adult patients, necessitating more forethoughtful study and understanding of the mechanisms behind CRC and the development of the new diagnostic and therapeutic strategies. Our research demonstrated that SPT6 synergized with staphylococcal nuclease and Tudor domain containing 1 (SND1) to promote CRC progression by targeting human telomerase reverse transcriptase (hTERT) and put forward that inhibiting SPT6‐SND1‐hTERT axis may create a therapeutic vulnerability in CRC.

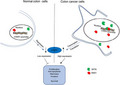

**Abbreviations**
AOM/DSS: azoxymethane/dextran sulfate sodium
APC: APC regulator of WNT signaling pathway
CRC: colorectal cancer
CSCs: cancer stem cells
EMT: epithelial–mesenchymal transition
hTERT: human telomerase reverse transcriptase
KRAS: KRAS Proto‐Oncogene, GTPase
SD: standard deviation
shRNA: short hairpin RNA
siRNA: small interference RNA
SND1: staphylococcal nuclease and tudor domain containing 1
SPT6: histone chaperone and transcription elongation factor

## 1 Introduction

As the third most common cancer worldwide, colorectal cancer (CRC) possesses high morbidity and mortality rate. Approximately, 1.2 million new CRC cases are diagnosed each year [1, 2]. With early screening, surgery, radiation, and/or chemotherapy, CRC can be effectively treated. Nevertheless, the incidence of CRC is still continuously rising among young adult patients with averaged age of under 45 and ~ 25% of patients suffer from systemic metastases, most frequently metastasizing to liver and lung [3], and possess very poor prognosis. Thus, considering the complexity, real‐time variability, and intratumoral heterogeneity during CRC occurrence and development, plus individual differences, the current knowledge about predictive biomarkers and prognostic factors of CRC and the treatment options available are still relatively limited, necessitating more forethoughtful study and a better understanding of the underlying mechanisms behind CRC and the development and improvement of the new diagnostic and therapeutic strategies [4, 5, 6, 7].

It is well established that telomeres stabilize DNA by protecting the ends of chromosomes, although they are progressively shortened with each cell division. Telomere length is kept by telomerase, which is a multisubunit complex and can amplify and add the telomere repeat TTAGGG to the telomere end. As the catalytic subunit of telomerase, human telomerase reverse transcriptase (hTERT) plays a key role in determining telomerase activity. hTERT is normally suppressed in somatic cells but is reactivated in more than 90% of human cancers, resulting in the replicative immortality and aggressive phenotype of cancer cells by preventing cell senescence and death caused by chromosome instability [8, 9, 10, 11]. Numerous studies have demonstrated the involvement and promotion of hTERT in tumor progression by regulating various signaling pathways, cell factors, and cell functions [12, 13, 14, 15, 16]. In CRC, the expression of hTERT was shown to determine the metastatic phenotype of cancer cells [17] and to be related to the worse survival rates in patients with hepatic colorectal metastases after resection [18]. Even more, combined with APC regulator of WNT signaling pathway (APC), KRAS, and B‐Raf proto‐oncogene, serine/threonine kinase, hTERT has been emerged as one of the representative oncogenes to drive CRC [19]. However, how the hTERT is silenced in normal cells but overactivated in tumor cells remains poorly defined and deserves to be better explored.

Given that the regulation of the transcripts is exerted at multiple levels, including transcription initiation, transcription extension, termination, splicing, and RNA stabilization, the transcriptional landscape is known to be extraordinarily complicated. The specificity of transcription initiation is partially decided by transcription elongation factors. Among these factors, Suppressor Of Ty 6 Homolog (SPT6) is a conserved protein, which can directly interact with RNA polymerase II (RNAPII) [20, 21], histones [22], and the essential factor Iws1 [23, 24]. During the regulation of transcription, SPT6 regulates the structure of chromatin through histone modifications [25] and controls the fidelity of transcription initiation [26]. Recently, it is reported to interact with the histone methyltransferase nuclear receptor binding SET domain protein 2 to facilitate interferon‐induced transcription [27]. Although SPT6 plays a key role in the transcription regulation, its precise function, and the corresponding mechanisms in cancer initiation and development remain unclear, including CRC. In regard to its role as a specified transcriptional factor to regulate the expression of hTERT, it is entirely unknown.

In this work, based on the synthesized biotinylated hTERT promoter probe, we exploited pull‐down and mass spectrometry techniques to screen the possible binding proteins of hTERT promoter in CRC cells and identified one of them was SPT6. The regulation of SPT6 on hTERT expression and further on tumor growth was investigated and confirmed *in vitro* and *in vivo*. Moreover, considering the complexity of transcriptional regulation and the prediction failure of the direct binding site at hTERT promoters for SPT6, we further explored the underlying synergistic factor of SPT6 in regulating hTERT expression and confirmed such synergy to control hTERT transcription and tumor survival *in vitro*, *in vivo*, and clinically.

## 2 Materials and methods

### 2.1 Cell lines

Cell lines (CCD841, CCC‐HIE2, RKO, LoVo, DLD1, SW620, SW480) were obtained from American Type Culture Collection (Manassas, VA, USA). Cell line FHs 74 Int was purchased from typical culture collection of the Chinese Academy of Sciences committee on cell/stem cell libraries. CCD841, RKO, and SW620 were cultured in Dulbecco's modified Eagle's medium (DMEM) supplemented with 10% FBS. CCC‐HIE2 was cultured in DMEM supplemented with 20% FBS. LoVo, DLD1, and SW480 were maintained in RPMI‐1640 medium containing 10% FBS. FHs 74 Int was maintained in Hybri‐Care Medium with 10% FBS and 30 ng·mL^−1^ rhEGF. All the cell lines mentioned above were cultured in a humidified atmosphere with 5% CO_2_ at 37 °C

### 2.2 Antibodies

The antibodies against SPT6 (sc‐393938), staphylococcal nuclease and Tudor domain containing 1 (SND1; sc‐271590), cytochrome *C* (sc‐13561), and PCNA (sc‐56) were purchased from Santa Cruz (Dallas, TX, USA). The antibodies against vimentin (#5741), snail (#3879), slug (#9585), Oct4 (#2750), EpCAM (#2929), Caspase‐3 (#9662), c‐Caspase‐9 (#9505), GSK‐3β (#9323), p‐PTEN (#9554), p‐PDK1 (#3438), p85 (#4228), MMP‐9 (#3852), p‐GSK3β (#5558), IκBα (#4812), p‐IκBα (#2859), p‐Erk (#9102), and Erk (#9101) were purchased from Cell Signaling Technology (Beverly, MA, USA). The antibodies against β‐actin (20536‐1‐AP), PARP (13371‐1‐AP), E‐cadherin (20874‐1‐AP), N‐cadherin (22018‐1‐AP), CD133 (18470‐1‐AP), and CD44 (15675‐1‐AP) were purchased from Proteintech Group (Wuhan, Hubei, China). The antibody against TERT (ABE2075) was purchased from Millipore (Burlington, MA, USA).

### 2.3 Plasmids transfection

SPT6‐targeting short hairpin RNAs (shRNAs) and control shRNA plasmids were purchased from GeneCopoeia. The infection was mediated by lentivirus following the kit's protocol (Lenti‐Pac HIV expression packaging kit, GeneCopoeia). In brief, the package cells were transfected with plasmids/EndoFectin Lenti complex for 48 h. Then, the supernatants were collected, centrifuged, and filtered. After determining the viral titer by serial dilutions, the virus was used to infect target cells. The stably transduced cells were finally obtained using puromycin selection.

### 2.4 siRNAs and transfection

In this study, cells were transfected with small interference RNA (siRNA; GenePharma, Suzhou, China). The sequences of each siRNA pair were as follows: SPT6siRNA‐1: 5′‐GAGCUGAGCUGUCGAUAUATT‐3′ and 5′‐UAUAUCGACAGCUCAGCUCTT‐3′; SPT6siRNA‐2: 5′‐GCCGCAUCAUGAAGAUUGATT‐3′ and 5′‐UCAAUCUUCAUGAUGCGGCTT‐3′; SND1siRNA‐1: 5′‐GGGAGAACACCCAGGATAA‐3′ and 5′‐ UUAUCCUGGGUGUUCUCCC‐3′; and SND1siRNA‐2: 5′‐ CAGCAAAGGTCTAGCCACA‐3′ and 5′‐ UGUGGCUAGACCUUUGCUG‐3′. The sequence of negative control siRNA was as follows: 5′ UUCUCCGAACGUCUCACGUTT and 3′‐ACGUGACACGUUCGGAGAATT. siRNA duplexes were transfected into cells using lipoplex made in our laboratory.

### 2.5 ChIP assay

RKO, LoVo, DLD1, SW620, and SW480 cells were fixed with 1% formaldehyde, collected, and sonicated with an ice bath to shear the DNA into 200–500 bp fragments. The cell lysate was subjected to incubation with the antibodies against SPT6 or nonspecific rabbit IgG and protein A/G agarose beads for immunoprecipitation. The immunoprecipitated DNA was finally used as templates to amplify the hTERT promoter fragment with 220 bp *via* PCR. The sequence of the primers was as follows: Up: 5′ ‐ ACCCTGGGAGCGCGAGCGGC ‐ 3′, Down: 5′ ‐ GGGGCGGGGTCCGCGCGGAG ‐ 3′. The amplified products were detected by GoldView staining (Meilunbio, Dalian, China, MB0078) after electrophoretic separation on 1% agarose gel.

### 2.6 Pull‐down assay

Pull‐down assay was carried out as previously described [28]. In brief, the bio‐labeled double‐stranded oligonucleotide probes', which correspond to the hTERT promoter sequence (−459 to +43) were synthesized by PCR using biotin‐labeled primers from TAKARA Company. The sequences of the primers used was: Up: 5′ ‐ (Biotin) ACCTGGAGGCAGCCCTGGGTCT‐3′, Down: 5′ ‐ (Biotin) CAGCAGGACGCAGCGCTGCCTG‐3. The nucleus proteins (400 μg) from cell lysate were mixed with double‐strand biotinylated hTERT probes (4 μg), streptavidin agarose beads (50 μL) in 500 μL PBSI buffer (0.5 mm PMSF, 10 mm NaF, 25 mm β‐glycerophosphate) and rotated for 4 h at RT. The beads were centrifuged, washed with PBSI buffer twice, and then were resuspended by loading buffer and boiled at 100 °C for 10 min. The supernatant was analyzed by western blot.

### 2.7 FACS assay

FACS assay was performed according to the protocol reported in the previous study [29]. Briefly, the detection of cell apoptosis was based on FACS analysis by FITC‐AV/PI staining. Cells were plated in six‐well plates and transfected with siRNAs. Forty‐eight hours later, the cells were harvested, washed with PBS, and centrifuged at 300 ***g*** for 3 min. The cells were resuspended in 500 μL binding buffer and stained with 5 μL Annexin V‐FITC (AV), 5 μL propidium iodide (PI) using an Annexin V‐FITC/PI staining kit (KeyGene BioTech, Nanjing, Jiang su, China). The status of cell apoptosis was analyzed by flow cytometry (BD ACCURI C6).

### 2.8 Telomerase activity assay (Telomeric Repeat Amplification Protocol)

Telomerase activity was measured using the TeloTAGGG Telomerase PCR ELISA Kit (Cat: 11854666910; Roche, Basel, Switzerland) according to the manufacturer's instructions. SW620 or RKO cells were seeded into six‐well plate and transfected with spt6 or snd1 specific siRNAs or control siRNAs. Forty‐eight hours later, the treated cells (about 2 × 10^5^) were collected for the assay. Briefly, telomerase adds telomeric repeats to the specific primer, and these elongation products were amplified by PCR. The PCR products were used for the detection of ELISA. Absorbance at 450 nm was normalized to the control treatment group, which was set at 100%.

### 2.9 qPCR for telomere length measurement

SW620 or RKO cells were seeded into six‐well plate and transfected with spt6 or snd1 specific siRNAs or control siRNAs. Forty‐eight hours later, genomic DNA from different treated cells (1 × 10^6^ cells) was isolated using TIANamp Genomic DNA Kit (Cat: #DP304‐02, TIANGEN, Beijing, China) according to manufacturer's protocol. Average telomere length was measured from total genomic DNA by using the qPCR method previously described [30]. Briefly, qPCR was performed using 14 ng of genomic DNA, different concentrations of reverse and forward primers (100 nm for tel1b, 900 nm for tel2b, 300 nm for hbg1, and 700 nm for hbg2) and TransStart Top Green qPCR SuperMix (Cat: AQ131, Transgen). The sequence of the primers used are: telomeric repeats (T) tel1b: 5′‐ GGTTTTTGAGGGTGAGGGTGAGGGTGAGGGTGAGGGT‐3′, tel2b: 5′‐ TCCCGACTATCCCTATCCCTATCCCTATCCCTATCCCTA‐3′; human beta‐globin hbg1: 5′‐ ‐GCTTCTGACACAACTGTGTTCACTAGC‐3′, hbg2: 5′‐ CACCAACTTCATCCACGTTCACC‐3′. Mean TRF lengths were determined as described [31].

### 2.10 Acridine orange/ethidium bromide fluorescence staining

Acridine orange/ethidium bromide (AO/EB) assay was carried out as previously described [29]. The RKO and SW620 cells seeded into six‐well plates the day before were transfected with SPT6 specific siRNAs. Forty‐eight hours later, the cells were washed with PBS to discard the detached cells and were stained with a mixture of AO (Solarbio, Beijing, China) and EB (Solarbio). Briefly, AO and EB were mixed by 1 : 1 after being, respectively, diluted 100 times in PBS and 250 μL such mixed liquor was added into the cells in each well. After shaking several seconds, the liquor was removed and the staining results were observed and photographed by the inverted fluorescence microscope (Leica, Mannheim, Germany).

### 2.11 Colony forming assays

After siRNA transfection, about 1000 cells were subjected to growth in one well of the six‐well plate. Ten to 14 days later, the formed colonies were photographed by an inverted microscope (Lecia). After fixation and staining, colonies were scored using image pro plus software (Media Cybernetics, Silver Spring, MD, USA).

### 2.12 Wound‐healing assays

Colon cancer cells were seeded in six‐well plates and grown to subconfluence and transfected as indicated. The cell monolayer was scratched using a plastic pipette tip (200 μL) to create the linear wound. Then, the scratched cell monolayer was washed with PBS to remove debris and incubated in the medium with 2% FBS at 37 °C for 48 h. The migration of the cells toward the wound was photographed using a light microscope at 0 h and 48 h. The migration distance was evaluated using the image pro plus software (Media Cybernetics).

### 2.13 Transwell invasion assay

*In vitro*, invasive activity through a gel matrix was performed in 24‐well plates. Cell Culture Inserts (Corning Incorporated, New York, NY, USA) were coated with 70 μL of Matrigel and placed in each well of 24‐well plates filled with 500 μL complete medium. Cells were resuspended at a density of 1*10^4^ cells·mL^−1^, and 100 μL of such cell suspension was added into the upper insert. After 24 h, the cells that invaded to the lower surface of the filter were fixed using 4% paraformaldehyde, stained with 0.1% crystal violet and counted after removing the cells still existing on the upper surface of the filter using cotton swabs. Images of the stained cells were taken by an inverted microscope at 400× magnification.

### 2.14 Sphere‐forming assay

Cells after different treatments were digested into single cell, and 2000 cells were, respectively, seeded into 35‐mm nontreated cell culture dishes with continuous culture for 14 days in DMEM/F12 medium (HyClone, Logan, UT, USA) containing N2 supplement (GIBCO, Grand Island, NY, USA), B27 supplement (GIBCO), EGF (20 ng·mL^−1^), and bFGF (20 ng·mL^−1^). Then, the formed tumorspheres were photographed by an inverted microscope (Leica). The spheres larger than 50 μm at diameter were counted.

### 2.15 Cell viability assay

Five thousand cells were seeded into a well of a 96‐well plate and cultured overnight. Then, the cells were transfected with siRNA using lipoplex made in our laboratory. After 48 h, 100 μL medium containing MTT was added into single well and cultured for 4 h. Then, MTT was discarded and 150 μL DMSO was added into each well to dissolve the crystal. Finally, the OD value at 490 nm was measured.

### 2.16 Western blotting

Cell lysates were prepared by the addition of 100 μL lysis buffer with Cocktail (Selleck, Houston, TX, USA ) followed by incubation on ice for 30 min. Then, the lysate was centrifuged at 4 °C with 14 000 ***g*** for 20 min. The supernatant was collected, and the protein concentration was determined *via* the BCA protein assay kit (Thermo Fisher, Waltham, MA, USA). Thirty microgram proteins for each sample were separated using SDS/PAGE and then electrotransferred onto the polyvinylidene difluoride (PVDF) membranes for immunoblotting. After incubation with the antibodies of the target proteins, the signals were detected by chemiluminescence.

### 2.17 RT‐PCR

Total RNA was extracted from cells using Trizol (Invitrogen Life Technologies, Carlsbad, CA, USA ) following the manufacture's protocol, and the concentration was measured. Then, the cDNA was synthesized according to the given protocol of Prime‐ScriptTM RT‐PCR Kit (TaKaRa). The sequences of the primers are as follows: SPT6 (forward ‐ 5′GCTTCAGACAGTTATCTTCAGGC3′, reverse ‐ 5′TCCAGGCGGTCATCAAAAGA3′); TERT (forward ‐ 5′AGGCTAACGGAGGTCATCG3′, reverse ‐ 5′GGCTGGAGGTCTGTCAAGGTA3′); SND1 (forward ‐ 5′GACTACGGCAACAGAGAGGT3′, reverse ‐ 5′TTGGGCATTCAGGTATTCTGTGA3′); β‐actin (forward ‐ 5′GGCACCCAGCACAATGAA3′, reverse ‐ 5′TAGAAGCATTTGCGGTGG3′).

### 2.18 Dual‐luciferase reporter assay

Luciferase activities were determined using the Dual‐Luciferase Assay System (Promega, Madison, WI, USA). The results were expressed as a ratio of firefly luciferase (Fluc) activity to Renilla luciferase (Rluc) activity. Data were collected from at least three experiments, and the standard deviation was calculated.

### 2.19 Immunohistochemistry staining

Tumors were dissected, fixed in 10% formalin overnight, embedded in paraffin, and then incised to 4‐μm‐thick section for the staining. Immunohistochemistry (IHC) staining was conducted according to the protocols of the used DAB Kit (Origene, Rockville, MD, USA). In brief, the sections were heated for half an hour at 65 °C. The paraffin was discarded with xylene, and the sections were rehydrated with ethanol. After antigen retrieval, the sections were blocked with goat serum for 60 min at room temperature, and then, the primary antibodies, respectively, against CD44, hTERT, PCNA, E‐cadherin, N‐cadherin, SPT6, and SND1 was added to the slide with indicated dilution ratio and incubated overnight at 4 °C. Hematoxylin was used to stain the nucleus. The visualized signal was developed with 3, 3′‐diaminobenzidine (DAB), and the protein expression level was scored based on the staining color.

### 2.20 Immunofluorescence assay

Immunohistochemistry assay was carried out as previously described [28]. RKO, LoVo, and SW620 cells grown on chamber sliders in each well of a six‐well plate were fixed with 4% paraformaldehyde for 10 min at RT and permeabilized with PBST (PBS with 0.2% Triton X‐100) for 5 min. Then, the cells were blocked with BSA for 30 min followed by incubation with the antibody, respectively, against cytochrome *C*, SPT6 or SND1 diluted in PBS with 1% BSA at 4 °C overnight. After washing with PBS for three times with 10 min each time, Alexa Fluor 488 or Alexa Fluor 555‐conjugated secondary antibodies were added to the cells with continuous incubation for 30 min. The nucleus was stained with 4′, 6‐diamidino‐2‐phenylindole (DAPI). After five times of washing with PBS, the images were finally visualized by confocal laser microscope (Leica DM 14000B). The percentage of positive staining was divided into five levels. 0 for no positive staining (< 5%), 1 for positive staining (5–25%), 2 for positive staining (26–50%), 3 for positive staining (51–75%), and 4 for positive staining (> 75%). The color intensity was divided into four levels. 0 for Colorless, 1 for pale yellow, 2 for brown, and 3 for tan. The final score of IHC staining is multiplied by these two parts. When the tissue isomerism in slices appeared, the score for each part was independent, and the final score was obtained by summing up these different parts. Concerning clinical samples, the displayed score for the expression level of target genes is further divided into two levels: low (1) and high (2). Low represents the final score is 0–7, and high represents the final score is 8–12.

### 2.21 Immunoprecipitation for MALDI‐TOF MS assay

Protein was extracted from the cells. The cell lysate containing 400 μg total proteins was precleaned using the Protein A/G beads firstly. Then, the supernatant after centrifugation was collected and added to the recommended amount of antibody to incubate overnight. Protein A/G agarose bead slurry was added next for a continuous incubation overnight. The immunocomplexes are collected and boiled with the loading buffer. The supernatant was loaded onto the SDS/PAGE gel to separate the proteins. After silver stain, the bands having differences were cut and sent to do the mass spectrometry analysis.

### 2.22 Animal experiments

All animal maintenance and operations were carried out following the National Institute of Health Guide for the Care and Use of Laboratory Animals, with the approval of the Animal Research Committee of Dalian Medical University. Male BALB/c nude mice, 4–5 weeks old, were purchased from Charles River Laboratories. Animals were randomly assigned to different groups when they reached 6–7 weeks old and received the corresponding treatments. To evaluate the impact of SPT6 expression level on the tumor metastasis *in vivo*, SW620 cells (5*10^6^) with or without stable knockdown of SPT6 were resuspended in PBS (100 μL) and then injected into the tail vein of the mice (three mice for each group). Lung metastasis was detected using *Maestro 2.10 IN‐VIVO IMAGING SYSTEM* 40 days after injection. To evaluate the impact of SPT6 expression level on tumor growth, LOVO cells (5*10^6^) resuspended in PBS (100 μL) were injected into the left flank of each mouse. Fourteen days after injection, when the palpable tumors were observed, control siRNAs, or SPT6‐specific siRNAs encapsulated by liposome were injected intratumorally once every 3 days, and the tumor volume was calculated every 2 days. To evaluate the synergistic impact of SPT6 and SND1 on the tumor progression *in vivo*, LoVo cells (5*10^6^) with or without stable overexpression of SPT6 and/or SND1 shRNAs were resuspended in PBS (100 μL) and injected into the left flank of each mouse. Tumor diameter was measured by caliper 2–3 times per week and tumor volume in mm^3^ was calculated as (length*width^2^)/2. Tumor weight was recorded after the mice were sacrificed. The induction of the azoxymethane/dextran sulfate sodium (AOM/DSS) model using C57BL/6 male mice was carried out as previously described [32]. The mice were divided into three groups: five mice without AOM/DSS induction, five mice with AOM/DSS induction for 14 days, and 10 mice with AOM/DSS induction for 107 days.

### 2.23 Statistical analysis

The specific statistical tests were carried out using graphpad prism version 7.0 (GraphPad Software, Inc., San Diego, CA, USA). Statistical analysis for the immunohistochemistry staining and clinical data was performed using IBM SPSS statistics 24 (SPSS, Inc., Chicago, IL, USA). *P* < 0.05 was considered to be significant.

## 3 Results

### 3.1 SPT6 transcriptionally regulates hTERT in colon cancer

**Fig. 1:**
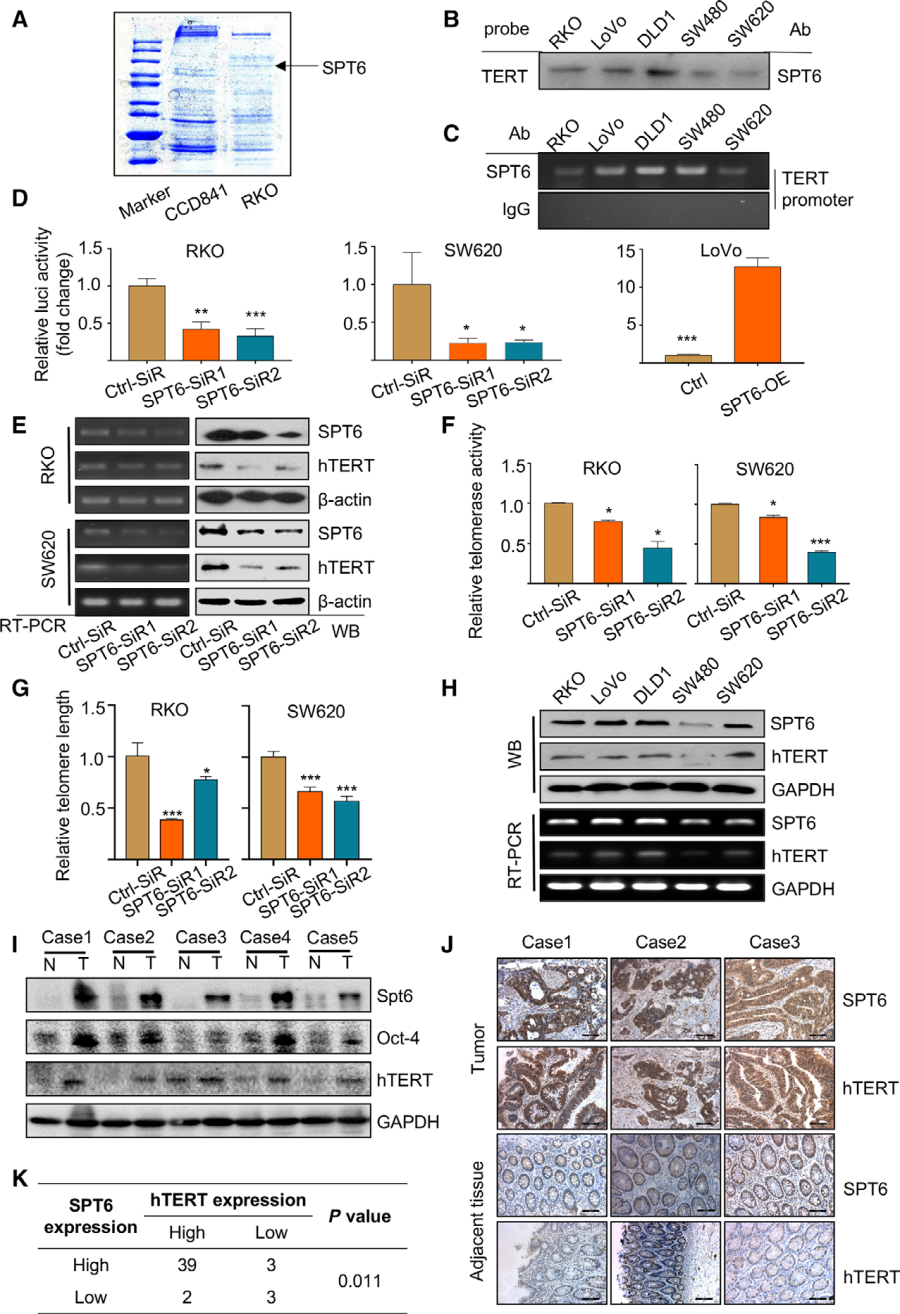
SPT6 transcriptionally regulates hTERT in colon cancer cells. (A) Coomassie blue staining of the possible binding proteins at hTERT promoter in normal colon cell line and the colon cancer cell line and SPT6 was identified as one of them. (B) Western blot determination of the interaction between SPT6 and the promoter of hTERT in five colon cancer cell lines after Pull‐down assay. (C) ChIP assay of the interaction between SPT6 and the promoter of hTERT in five colon cancer cell lines. (D) Dual‐luciferase reporter assay driven by hTERT promoter in colon cancer cells with SPT6 knockdown (RKO and SW620) or overexpression (LoVo). Data are presented as mean ± SD (*n* = 3). ∗*P* < 0.05, ∗∗*P* < 0.01, ∗∗∗*P* < 0.001. (E) RT‐PCR and WB assay of hTERT expression in colon cancer cells upon SPT6 knockdown. (F) The knockdown of SPT6 suppressed the telomerase activity in colon cancer cell lines. Data are presented as mean ± SD (*n* = 3). ∗*P* < 0.05, ∗∗∗*P* < 0.001. (G) The knockdown of SPT6 shortened telomere length in colon cancer cell lines. Data are presented as mean ± SD (*n* = 3). ∗*P* < 0.05, ∗∗∗*P* < 0.001. (H) RT‐PCR and WB assay of the expression of SPT6 and hTERT in different colon cancer cell lines. (I) The expression of SPT6 and hTERT in the tumor tissues (T) and the adjacent noncancer tissues (N) from patients with CRC was evaluated through WB. Representative pictures are shown. (J) The expression of SPT6 and hTERT in the tumor tissues (T) and the adjacent noncancer tissues (N) from patients with CRC was evaluated through IHC. Representative pictures are shown. Scale bars represent 100 μm. (K) The expression correlation between SPT6 and hTERT was shown according to the quantification of IHC.

To explore the related transcriptional regulation mechanism of hTERT in colon cancer development, we firstly designed biotin‐labeled hTERT promoter probes and performed Pull‐down/MS assay in different colon cancer cell lines. A specific transcription elongation factor, SPT6, was identified in the pulled down protein complexes (Fig. 1A). Western blot analysis for the pulled down proteins in different colon cancer cells and normal intestinal epithelial cells verified the chosen protein was SPT6 and its binding to the promoter of hTERT was tumor specific (Fig. 1B, Fig. S1A). To further confirm the binding of SPT6 at the promoter of hTERT, a ChIP assay was applied (Fig. 1C). hTERT promoter sequence corresponding to −378 to −159 was, respectively, amplified in different colon cancer cells upon SPT6 antibody treatment, but nearly not upon IgG treatment. Since SPT6 was observed to interact with the hTERT promoter, we next detected the possibility of SPT6 in regulating hTERT expression. Dual‐luciferase reporter assay, RT‐PCR and WB assay were carried out within different colon cancer cell lines. The results proved that the knockdown of SPT6 decreased hTERT promoter (−459 to +40)‐driven luciferase activity, while its overexpression caused opposite effect (Fig. 1D). SPT6 silencing also downregulated −321 to +40 and −234 to +40, but not −144 to +40 fragment within hTERT promoter‐driven luciferase expression (Fig. S1B), demonstrating the precise anchoring of SPT6 at −234 to −144 fragment of hTERT promoter region. RT‐PCR and WB assay similarly showed that the silencing of SPT6 significantly downregulated the expression of hTERT at mRNA and protein level (Fig. 1E, Fig. S1C). Besides, the Telomeric Repeat Amplification Protocol assay and telomere length assay further confirmed the downregulation of the telomerase activity and telomere length mediated by SPT6 knockdown (Fig. 1F,G).

To further explore the potential regulatory relationship between SPT6 and hTERT in colon cancer progression, we measured the expression of SPT6 and hTERT in colon cancer cell lines, clinical tissue samples from patients with colon cancer (Table 1), and online databases (CCLE, Cancer Cell Line Encyclopedia). The results indicated that both SPT6 and hTERT were highly expressed in tumor tissues compared with normal tissues and their expression showed a remarkable positive correlation, not only in different colon cancer cells but also in tumor tissues (Fig. 1H–K, Fig. S1D). The differential expression of SPT6 and hTERT between normal and tumor tissues and their expression correlation was further confirmed based on the analysis of TCGA data and microarray data analysis from human CRC (R2: Genomics Analysis and Visualization Platform; Fig. S1E,F). All the results above demonstrated the expression regulation of hTERT by SPT6 and their potential relationship in colon cancer cells and tissues.

**Table 1:** Score of IHC staining for the expression of SPT6, SND1, and hTERT based on clinical tissue samples from patients with colon cancer.

Patient ID	Tissue	Sex	Age	Size	TNM	Metastasis	Differentiation	Expression of hTERT	Expression of SPT6	Expression of SND1
1	Tumor	Female	61	6.5	IIb	N0	Mid	2	2	2
2	Tumor	Male	36	4	IIIc	N2a	High	1	2	2
3	Tumor	Male	50	5	IIb	N0	High	1	1	2
4	Tumor	Male	74	10	IIb	N0	High	1	1	1
5	Tumor	Male	40	6.5	IIb	N0	Mid	1	1	1
6	Tumor	Male	57	4	IIb	N0	Mid	1	1	1
7	Tumor	Female	68	5	IIb	N0	Mid	1	2	1
8	Tumor	Male	80	3.5	IIIb	N1a	Mid	1	1	1
9	Tumor	Male	73	6	IIa	N0	Mid	1	1	1
10	Tumor	Female	71	3.5	IIIc	N2b	Mid	1	1	1
11	Tumor	Female	61	10	IIIb	N1a	Mid	1	1	1
12	Tumor	Male	52	6.5	IIIb	N1a	Mid	2	2	1
13	Tumor	Male	63	5.5	IIb	N0	High	1	2	2
14	Tumor	Male	62	7.5	IIb	N0	High	2	2	2
15	Tumor	Female	47	5.5	IIIc	N2a	High	1	1	2
16	Tumor	Male	58	2.5	IIc	N0	Mid	1	1	1
17	Tumor	Female	61	4.5	IIIb	N1a	mid	1	1	2
18	Tumor	Female	79	5	IIIc	N2a	Mid	1	2	1
19	Tumor	Female	59	5	IIIb	N1a	High	2	2	2
20	Tumor	Male	75	5	IIIb	N1a	Mid	1	1	1
21	Tumor	Male	56	5	IIb	N0	High	1	1	2
22	Tumor	Male	68	3	IIb	N0	Mid	2	2	2
23	Tumor	Male	49	9	IIb	N0	High	2	1	1
24	Tumor	Female	57	3.2	IIIb	N1a	Mid	1	1	2
25	Tumor	Female	65	4.5	IIIc	N2a	Mid	1	1	1
26	Tumor	Female	68	2.7	IIIb	N1b	Mid	1	1	2
27	Tumor	Male	75	4	IIIb	N1b	Mid	2	1	1
28	Tumor	Male	71	5	IIb	N0	Mid	1	1	1
29	Tumor	Female	51	6	IIb	N0	Mid	1	2	1
30	Tumor	Male	44	7.5	IIIb	N1b	Mid	1	2	2
31	Tumor	Male	62	5	IIb	N0	High	2	1	1
32	Tumor	Female	54	3.5	IIIb	N1a	Mid	1	1	1
33	Tumor	Male	74	4.5	IIb	N0	Mid	1	1	2
34	Tumor	Male	64	6	IIb	N0	High	2	2	2
35	Tumor	Female	55	6	IIIc	N2a	High	1	1	1
36	Tumor	Male	67	9	IIb	N0	High	2	1	1
37	Tumor	Female	64	8	IIIb	N1a	High	1	2	2
38	Tumor	Male	70	8	IIIc	N2a	Mid	1	2	1
39	Tumor	Male	51	5	IIIb	N1a	Mid	1	2	1
40	Tumor	Female	81	9	IIIb	N1b	High	2	2	2
41	Tumor	Male	72	4.5	IIb	N0	Mid	2	1	2
42	Tumor	Male	68	6	IIb	N0	Mid	1	2	1
43	Tumor	Male	58	1.5	IIb	N0	High	1	2	2
44	Tumor	Female	75	4	IIb	N0	High	2	1	2
45	Tumor	Female	84	6	IIb	N0	Mid	1	1	1
46	Tumor	Male	82	7	IIIc	N1	High	1	1	2
47	Tumor	Male	53	4	IIb	N0	High	1	1	1

### 3.2 SPT6 promotes the proliferation and metastasis of colon cancer cells *in vitro*

**Fig. 2:**
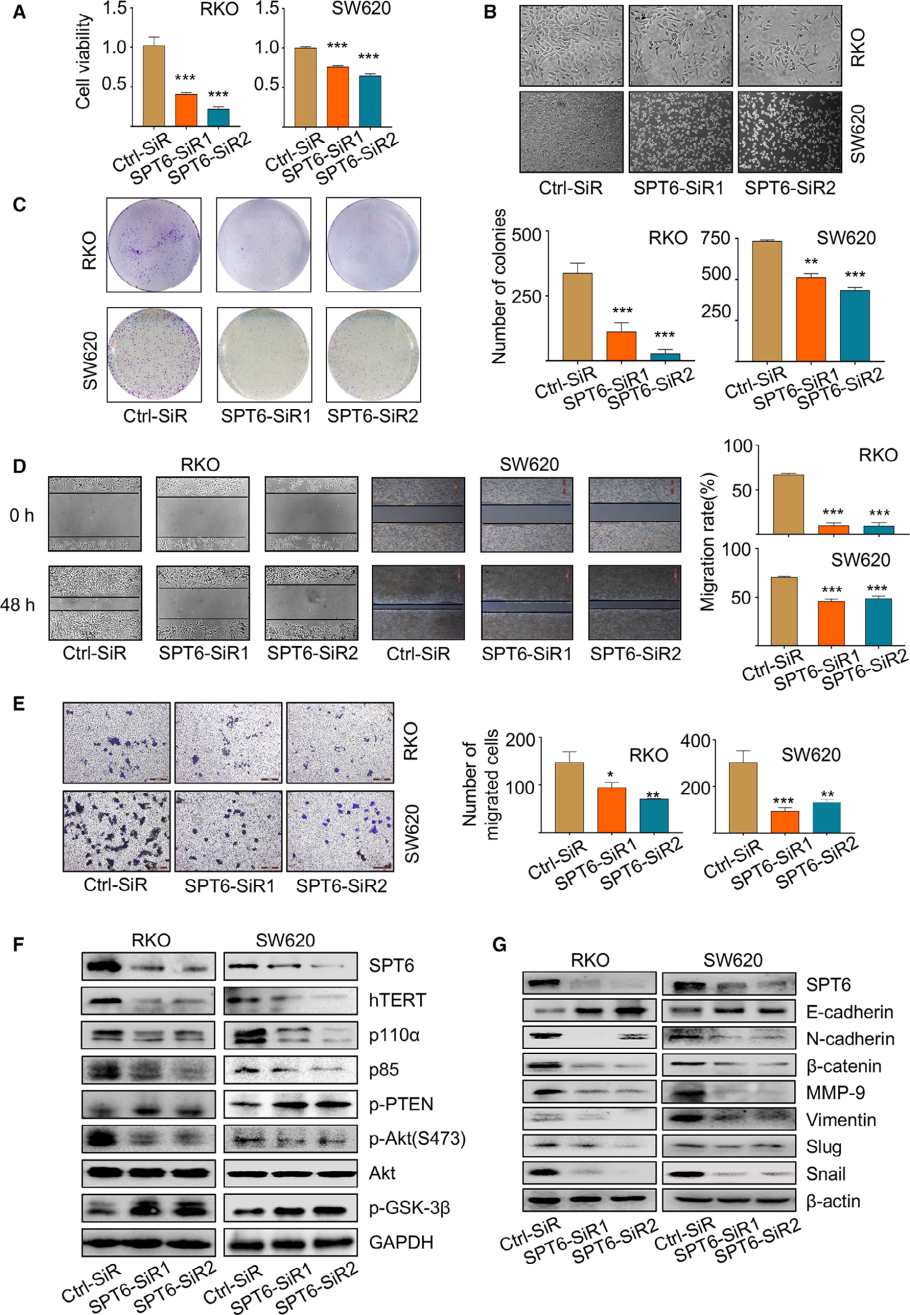
SPT6 promotes the proliferation and metastasis of colon cancer cells *in vitro*. (A) After knocking down SPT6, MTT assay was carried out to measure the cell viability in two colon cancer cell lines, RKO and SW620. ∗∗∗*P* < 0.001. (B) The impact of knocking down SPT6 on the morphology of colon cancer cells and the representative images are shown (*n* = 3). Scale bars represent 200 μm. (C) Colony forming assay was carried out in two colon cancer cell lines to evaluate the impact of knocking down SPT6 on the proliferation. Representative pictures are shown (*n* = 3). Scale bars represent 200 μm. Quantification of the colony forming assay was performed using image pro plus. Data are presented as mean ± SD (*n* = 3). ∗∗*P* < 0.01, ∗∗∗*P* < 0.001. (D) Wound‐healing assay was performed in two colon cancer cell lines to evaluate the impact of knocking down SPT6 on the migration. Representative pictures are shown (*n* = 3). Scale bars represent 200 μm. Quantification of the wound‐healing assay was performed using image pro plus. Data are presented as mean ± SD (*n* = 3). ∗∗∗*P* < 0.001. (E) Transwell assay in two colon cancer cells upon SPT6 knockdown. Representative pictures are shown (*n* = 3). Data are presented as mean ± SD (*n* = 3). ∗*P* < 0.05, ∗∗*P* < 0.01, ∗∗∗*P* < 0.001. (F) Western blot assay showed the impact on PI3K/Akt signaling pathway markers after knocking down SPT6 in two colon cancer cell lines. (G) Western blot assay showed the impact on EMT markers after knocking down SPT6 in two colon cancer cell lines.

To explore the functional impact of SPT6 in CRC cell lines, we knocked down the expression of SPT6 using its specific siRNAs in RKO and SW620 cells. MTT assay showed that SPT6 knockdown markedly inhibited cell viability (Fig. 2A) and caused significant changes in cell morphology (Fig. 2B). Moreover, the knockdown of SPT6 also obviously inhibited the colony forming ability and led to migrative and invasive arrest in colon cancer cells (Fig. 2C–E). Considering the critical role of PI3K/Akt signaling pathway in regulating cancer cell growth and survival, we next examined the expressions of the key proteins involved in this pathway, and in addition to the expression of tumor suppressor PTEN and GSK‐3β, the expressions of other proteins were decreased with the knockdown of SPT6 (Fig. 2F). Besides, consistent with the functional alterations in cancer cell metastasis, the expressions of the epithelial–mesenchymal transition (EMT) markers were also significantly changed upon SPT6 silencing. The level of E‐cadherin was upregulated, while the other proteins, including N‐cadherin, β‐catenin, MMP‐9, vimentin, slug, and snail, were downregulated (Fig. 2G). Thus, these findings collectively suggest the potential role of SPT6 as a proliferative and pro‐metastatic factor in colon cancer, very possibly relying on its regulation on hTERT.

### 3.3 SPT6 knockdown induces apoptosis, stemness arrest, and chemosensitivity improvement in colon cancer cells

**Fig. 3:**
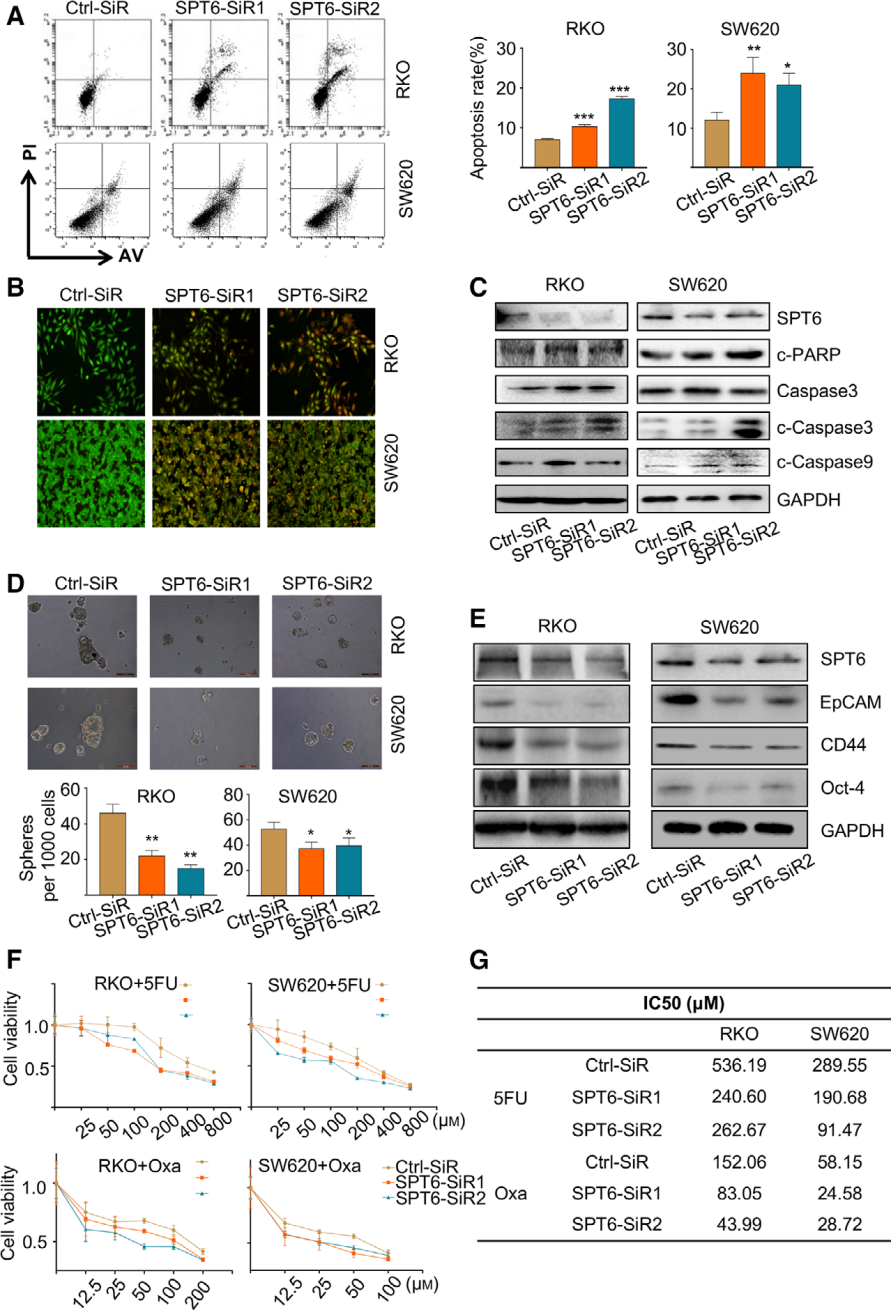
SPT6 knockdown induces apoptosis, stemness arrest and chemotherapeutic sensitivity improvement in colon cancer cells *in vitro*. (A) The apoptosis analysis of colon cancer cells upon SPT6 knockdown by flow cytometry based on FITC‐conjugated Annexin V/PI staining. The bar graph indicates the percentage of apoptotic cells. Data are presented as mean ± SD (*n* = 3). ∗*P* < 0.05, ∗∗*P* < 0.01, ∗∗∗*P* < 0.001. (B) The apoptosis analysis of colon cancer cells upon SPT6 knockdown by under a microscope following AO/EB staining. The cells with intact structures were stained green and were considered to be viable cells, whereas the cells with condensed yellow nuclei were identified as early apoptotic cells and those with condensed red‐orange chromatin were identified as late apoptotic cells. (C) Western blot assay showed the impact on apoptosis markers after knocking down SPT6 in two colon cancer cell lines. (D) The sphere‐forming assay was performed in colon cancer cells upon SPT6 knockdown as described in the methods section. Scale bars represent 500 μm. Quantification analysis of tumor spheres was performed using image pro plus. Data are presented as mean ± SD (*n* = 3). ∗*P* < 0.05, ∗∗*P* < 0.01. (E) Western blot assay showed the impact on stemness markers after knocking down SPT6 in two colon cancer cell lines. (F) Dose‐response curves of cell viability in RKO and SW620 cells transfected with control or SPT6 specific siRNAs and subsequently treated with varying concentrations of drugs for 48 h. (G) IC50 values of 5‐FU and Oxaliplatin in RKO and SW620 cells were treated as mentioned above.

There is accumulating evidence that hTERT favors an immortal phenotype by blocking apoptosis independently of its protective function at the telomere ends [33, 34, 35, 36]. Given the transcriptional regulation of hTERT by SPT6 approved above, we speculated that apoptosis blockade might similarly play a pivotal role in the proliferative function of SPT6. We first found that knockdown of SPT6 using its specific siRNAs promoted cell apoptosis compared with the control siRNA treatment (Fig. 3A,B). We also detected the change of cytochrome *C* release and the expressions of apoptosis‐related markers, and the results showed that SPT6 silencing led to more release of cytochrome *C* from mitochondria to cytoplasm and more accumulation of cleaved Caspase 9, Caspase 3, and PARP in colon cancer cells (Fig. S2A, Fig. 3C), confirming the promotability of apoptosis upon SPT6 knockdown.

Telomere maintenance was also reported to contribute to the self‐renewal of cancer stem cells (CSCs), and telomerase inhibition may work as a stem cell‐specific therapeutic approach [14, 37, 38]. We next examined the effect of SPT6 on stemness maintenance in colon cancer cells similarly by knocking down its expression using its specific siRNAs. Compared with the control siRNA treatment, SPT6 knockdown significantly suppressed the sphere‐forming ability of colon cancer cells accompanied by the expression decrease of stemness markers (Fig. 3D,E). Accordingly, considering the critical importance of chemotherapy in CRC treatments, and the major responsibilities of CSCs for chemotherapeutic resistance and tumor relapse, we thus investigated the role of SPT6 on the sensitivity of chemotherapeutic drugs. SPT6 knockdown significantly sensitized colon cancer cells, SW620, and RKO, to the treatments of chemotherapeutics, 5FU, and oxaliplatin, leading to a significant reduction in their IC50s (Fig. 3F–G, Fig. S2B). Taken together, these data further imply *in vitro* that SPT6 affects the functions of the colon cancer cells, including antagonizing apoptosis and increasing stem‐like properties, similarly and possibly relying on its regulation on hTERT.

To further confirm whether hTERT was involved in SPT6‐mediated functional regulation in colon cancer cells mentioned above, we overexpressed hTERT in RKO and SW620 cells with SPT6 silencing, respectively, and observed the cellular phenotype changes. The decreased cell viability and tumorsphere‐forming capacity upon SPT6 knockdown were all significantly reversed by hTERT overexpression (Fig. S2C,D), preliminarily demonstrating that SPT6 promoted colon cancer cell growth by targeting hTERT.

### 3.4 SPT6 knockdown inhibits tumor development and metastasis in mice

**Fig. 4:**
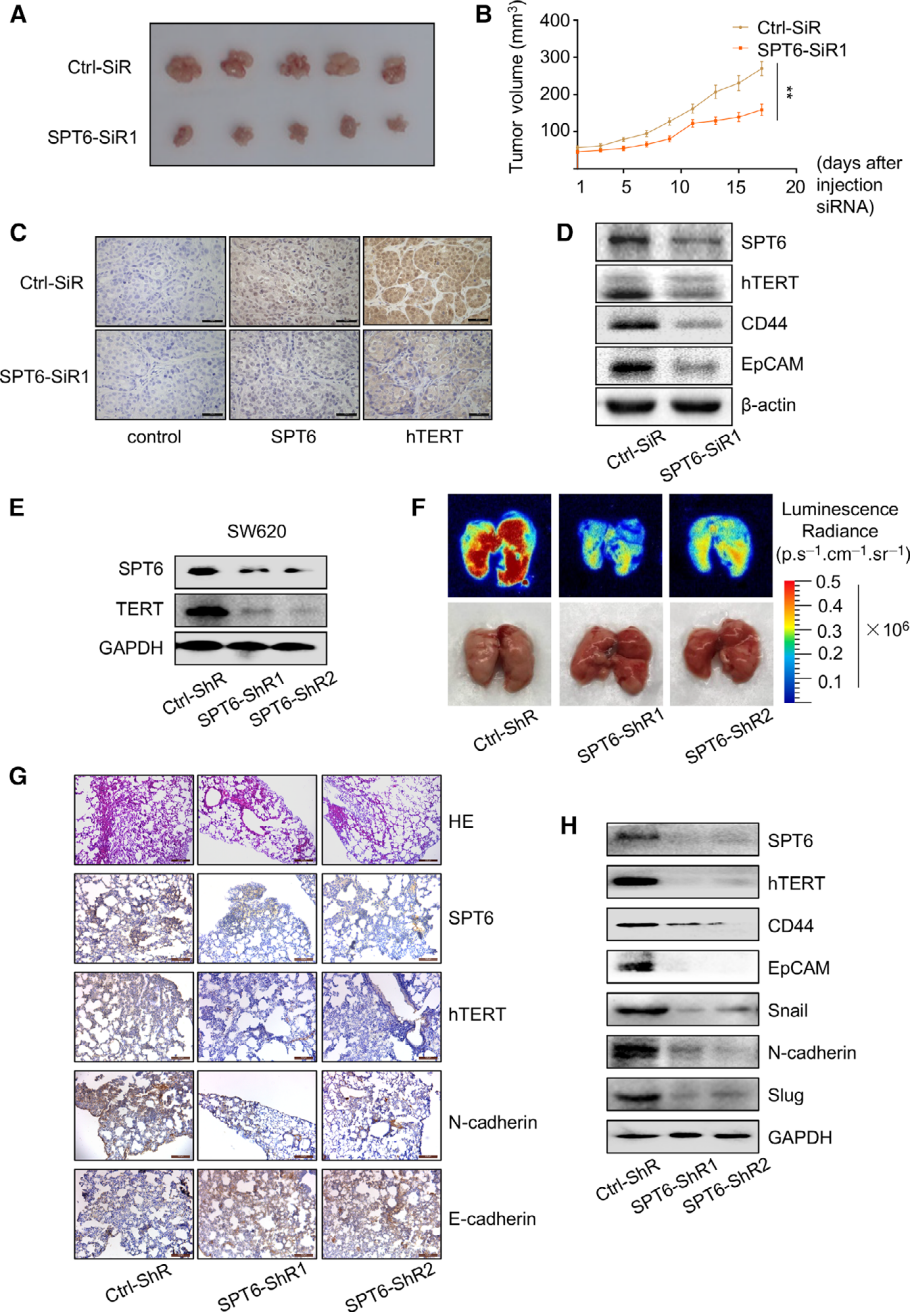
SPT6 knockdown inhibits tumor development and metastasis in mice. (A) The images of the tumors separated from the sacrificial mice on the 17th day after injection with control or SPT6 siRNA into the tumors. (B) The change of tumor volume upon knocking down SPT6 in the mice with xenograft of colon cancer cells. Data are presented as mean ± SD. ∗∗*P* < 0.01.(C) IHC staining of tumor sections collected from different treatment groups of mice at the end. Scale bars represent 100 μm. (D) Western blot assay of the expression of hTERT and stemness markers after knocking down SPT6 in tumors. (E) Western blot assay for the stable knockdown of SPT6 in SW620 cells. (F) The lung metastases of the mice assessed by *in vivo* fluorescence imaging and the corresponding apparent morphology imaging. (G) HE staining of the lung metastasis tissues in the mouse. Scale bars represent 200 μm and 100 μm). (H) Western blot assay of the invasion and stemness markers in lung metastasis tissues of mice.

To further investigate and confirm the role of SPT6 on hTERT expression and cancer development *in vivo*, the mouse model with xenografts of colon cancer cells was firstly established by injecting LoVo cells into BABL/c nude mice subcutaneously. When the tumor size reaches an average of ~ 50 mm^3^, the mice were randomly divided into two groups and SPT6‐siRNA or ctrl‐siRNA was, respectively, injected into the tumors of tumor‐bearing mice in each group every 3 days. The tumors were measured every 2 days for the duration of the study. Seventeen days later, the mice were sacrificed and the tumors were collected for analyzing. Compared with the ctrl group, tumor size, tumor growth, the expression of SPT6, hTERT, and stemness markers in tumor tissues was all significantly decreased in the group of SPT6 knockdown (Fig. 4A–D). Besides, we utilized a mouse model of metastasis by injecting SW620 cells with stable knockdown of SPT6 into mice through the tail veins to evaluate the role of SPT6 on tumor metastasis *in vivo* (Fig. 4E). Forty days after injection, the degree of lung metastasis was analyzed using *Maestro 2.10 IN‐VIVO IMAGING SYSTEM* and obvious suppression of metastasis was observed in the SPT6 knockdown group by comparison to the control group (Fig. 4F). Consistently, HE staining of lung tissue also showed the relatively small metastases of colon cancer cells with SPT6 knockdown (Fig. 4G). Furthermore, we also detected the expression change of EMT‐associated and stemness‐associated markers in lung tumor tissues upon SPT6 knockdown using IHC and WB assay, confirming that SPT6‐knocking down colon cancer cells possess comparatively low metastasis capacity and stemness sustainability (Fig. 4G,H). Based on the above evidence, we further confirmed the regulation of SPT6 on the expression of hTERT and the involvement of the SPT6/hTERT signaling axis in the development and metastasis of colon cancer.

### 3.5 SND1 recruits and interacts with SPT6 to co‐regulate hTERT expression and colon cancer cell survival *in vitro*

**Fig. 5:**
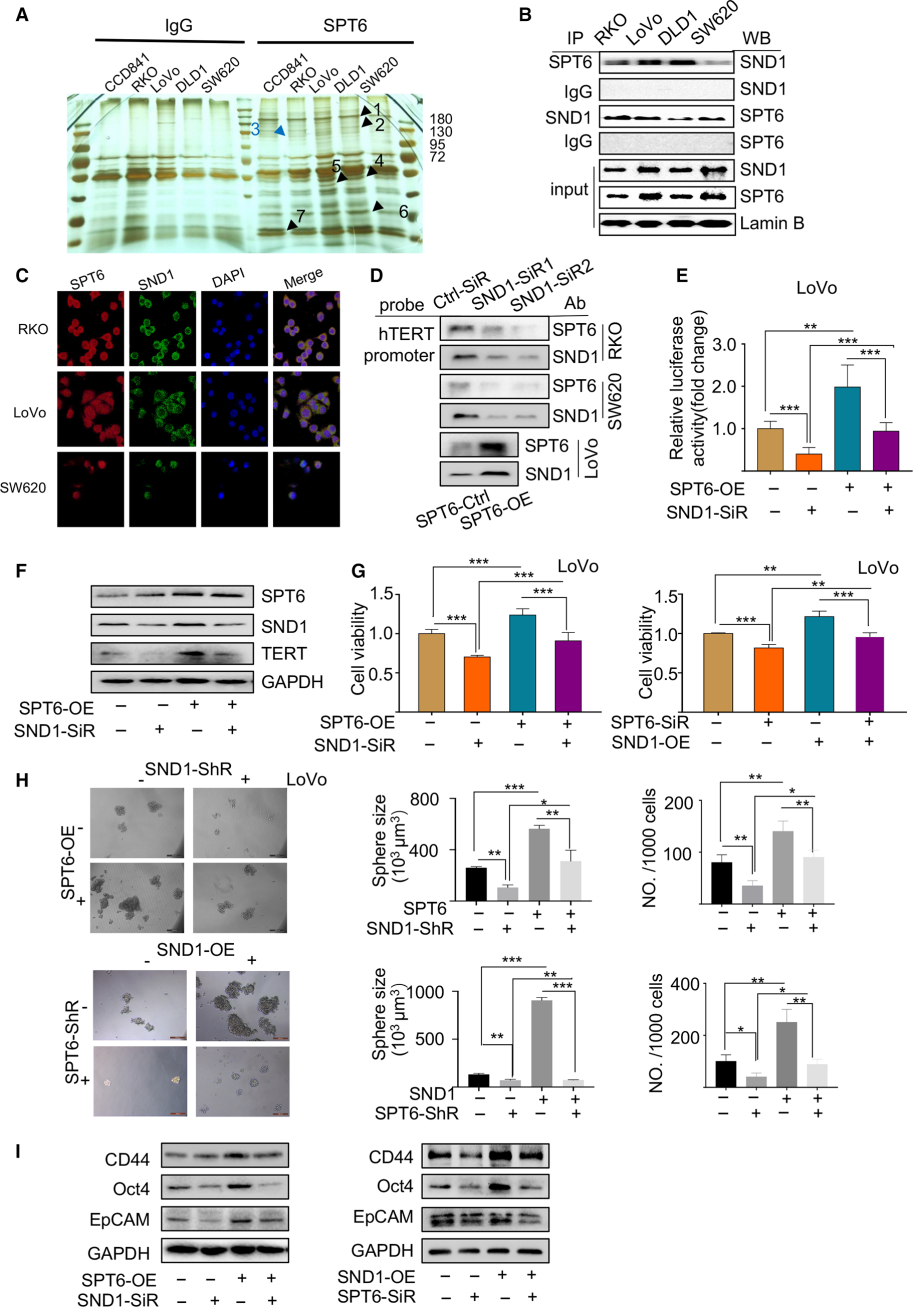
SND1 recruits and interacts with SPT6 to co‐regulate hTERT expression and colon cancer cell survival *in vitro*. (A) The silver staining of the proteins interacting with SPT6 in CCD841 (normal colon cell line), RKO, LoVo, DLD1, and SW620 (four colon cancer cell lines) immunoprecipitated by the antibody against SPT6. IgG was used as the control antibody. (B) Co‐IP results showing the interactions of SPT6 with SND1 in different colon cancer cell lines. (C) Immunofluorescence assay of the colocalization of endogenous SPT6 and SND1 in different colon cancer cells. Scale bar = 50 μm. (D) Pull‐down assay to evaluate the interaction of SPT6 and SND1 with the promoter of hTERT after knocking down SND1 in RKO and SW620 or overexpressing SPT6 in LoVo. (E) Dual‐luciferase reporter assay driven by hTERT promoter in LoVo cells without or with overexpression of SPT6 and with or without silencing of SND1. Data are presented as mean ± SD (*n* = 3). ∗∗*P* < 0.01, ∗∗∗*P* < 0.001. (F) Western blot assay for the rescue impact of knocking down SND1 during overexpressing SPT6 on the expression of hTERT in LoVo. (G) The cell viability assay to evaluate the recoverable effect of knocking down SND1 on the overexpression of SPT6 and knocking down SPT6 on the overexpression of SND1. Representative values of cell viability are represented by the mean percent of control ± SD (*n* = 3). ∗∗*P* < 0.01, ∗∗∗*P* < 0.001. (H) The sphere‐forming assay in LoVo cells without or with overexpression of SPT6 and/or silencing of SND1. Scale bars represent 500 μm. Quantification of spheres was performed using GraphPad Prism. Data are presented as mean ± SD (*n* = 3). ∗*P* < 0.05,**P < 0.01. (I) Western blot assay on the expression of stemness markers in LoVo cells without or with overexpression of SPT6/SND1 and/or silencing of SND1/SPT6.

Given the complexity of transcriptional regulation and tumor development [39, 40], we further explored the potential transcriptional factors, which may synergize with SPT6 to regulate the expression of hTERT and colon cancer progression. Through IP‐MS assay using one normal colon cell line and four colon cancer cell lines, we identified some factors cooperating with SPT6 specifically in colon cancer cells (Fig. 5A) and one of them was SND1. We re‐performed the IP assay and confirmed the interaction between SPT6 and SND1 (Fig. 5B). Immunofluorescent experiments also demonstrated the colocalization of SPT6 and SND1 in different colon cancer cells (Fig. 5C), further proving the possibility of their interaction. Based on the multiple functions of SND1, which is not only involved in the transcription and post‐transcription regulation but also closely related to the development of tumors [41, 42, 43, 44, 45], we hypothesized that SND1 may synergize with SPT6 to regulate the expression of hTERT and the development of tumors. To test this hypothesis, we firstly investigated the regulation of SND1 itself on hTERT expression. SND1 knockdown using its specific siRNAs led to the significant suppression of hTERT expression at mRNA and protein level (Fig. S3A,B) and also downregulated the activity of telomerase and telomere length (Fig. S3C,D). Additionally, we further observed the effect of SND1 expression on hTERT promoter‐driven luciferase activity. The knockdown of SND1 decreased hTERT promoter (−459 to +40)‐driven luciferase activity, while its overexpression produced opposite effect (Fig. S3E). Consistent with SPT6 silencing, SND1 knockdown also reduced −321to +40 and −234to +40, but not −144 to +40 fragment within hTERT promoter‐driven luciferase expression (Fig. S3F), illustrating the possibility that SND1 interacts with SPT6 to co‐anchor at −234 to −144 region of hTERT promoter. Furthermore, through Pull‐down assay, we observed the alleviated binding of SND1 at the promoter of hTERT when SND1 was knocked down (Fig. 5D), indirectly demonstrating the transcriptional regulation of SND1 on hTERT in colon cancer cells. What's more, knocking down SND1 suppressed the cell viability and tumorsphere formation, while the overexpression of hTERT reversed such suppression (Fig. S3G,H). In accordance with the above results, microarray data analysis of human CRC from GEO database revealed the significant positive correlation between SND1 and hTERT expression (Fig. S3I). Altogether, these results not only demonstrate the oncogenic role of SND1 in colon cancer cells by transcriptionally regulating hTERT, but also uncover the potential synergy between SND1 and SPT6 in co‐regulating hTERT.

Next, we explored the synergy produced by SPT6 and SND1 in regulating hTERT expression and colon cancer progression. We constructed LoVo cells with steady overexpression of SPT6 by lentivirus infection (Fig. S4A) and then transfected them with siRNA targeting SND1 or lentivirus carrying SND1 specific shRNAs. The markedly increased authoring of SND1 at hTERT promoter fragments upon SPT6 overexpression *via* pull‐down assay was observed, and vice versa, SND1 silencing led to the decreased binding of SPT6 at hTERT promoter accordingly (Fig. 5D). Besides, SND1 knockdown reversed the changes of the transcriptional activity of hTERT, the expression of hTERT, cell viability, tumorsphere‐forming ability and the expression of stemness‐associated markers in colon cancer cells mediated by SPT6 overexpression, and vice versa (Fig. 5E–I). To further confirm the synergy between SPT6 and SND1 in affecting tumor progression *in vitro*, we overexpressed or knocked down SPT6 and SND1 at the same time and evaluated their effects on the cell viability and sphere‐forming ability. More obvious inhibition upon their simultaneous silencing and more significant promotion upon their co‐overexpression were observed (Fig. S4B,C). In addition to these, we next explored the synergy between SPT6 and SND1 in regulating the sensitivity of tumor cells to chemotherapeutics. Consistently, the rescue impact was observed (Fig. S5A,B). Herein, at least based on the *in vitro* study, we concluded that SPT6 synergized with SND1 to co‐regulate hTERT expression and to be further involved in colon cancer progression.

### 3.6 SND1 knockdown impairs the promoted tumor growth mediated by SPT6 overexpression in mice carrying xenografts of human‐derived colon cancer cells

**Fig. 6:**
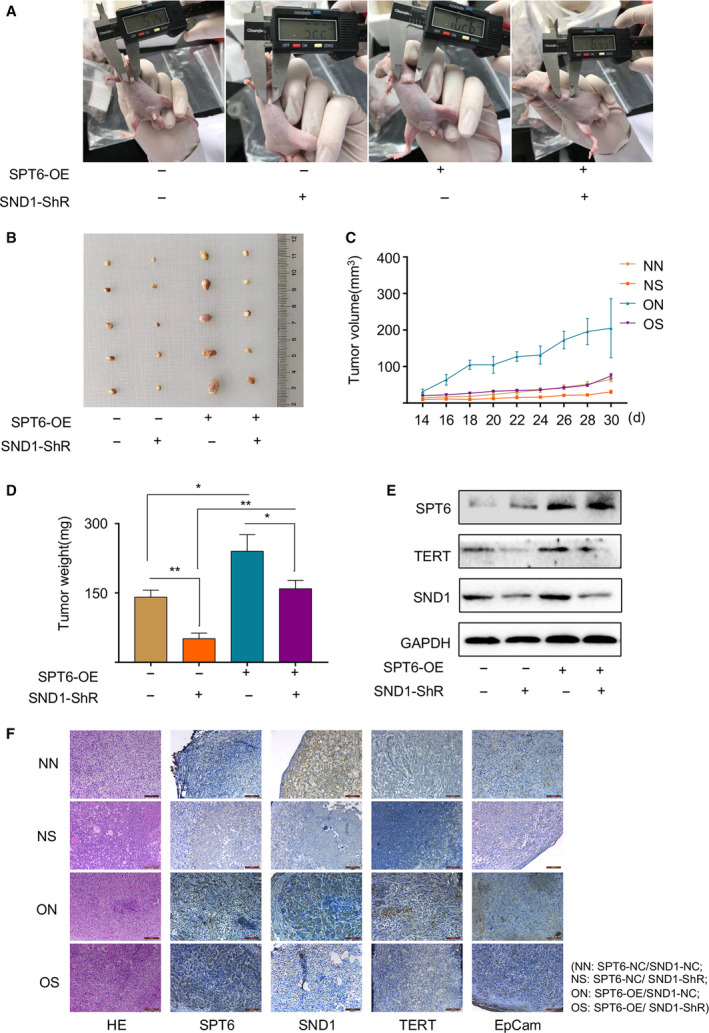
SND1 knockdown impairs the promoted tumor growth mediated by SPT6 overexpression in mice with xenografts of human colon cancer cells. (A) The representative photographs of mouse tumors formed by LoVo cell lines with or without stable overexpression of SPT6 and with or without silencing of SND1. (B) The images of the formed tumors in mice 30 days after injection of LoVo‐NN/NS/ON/OS cells (NN: SPT6‐NC/SND1‐NC; NS: SPT6‐NC/SND1‐ ShR; ON: SPT6‐OE/SND1‐NC; OS: SPT6‐OE/SND1‐ ShR). (C) The growth rates of tumors formed from LoVo‐NN/NS/ON/OS cells. After injection, tumor volumes were calculated every 2 days. (D) The tumor weights determined after harvesting the tumors. ∗*P* < 0.05, ∗∗*P* < 0.01. (E) Western blot assay of the expression of SPT6, SND1, and hTERT in the formed tumors. (F) HE and IHC staining for SPT6, SND1, TERT, and EpCam in mouse tumors. Scale bars represent 100 μm.

To further confirm the synergy of SND1 in the oncogenic function of SPT6 in colon cancer development, we next established xenografts of LoVo cells with stable SPT6 overexpression and SND1 knockdown or the corresponding control cells in mice by injecting them into the right flank of nude mice respectively. Two weeks after injection, the diameter of the formed tumors was measured and recorded every 2 days for 16 days. Group comparison showed that SPT6 overexpression facilitated the growth of tumors, while SND1 knockdown displayed the opposite effect. Of note, SND1 knockdown significantly reversed the promotion of tumor growth caused by SPT6 overexpression (Fig. 6A–D). Consistent with the observations *in vitro*, WB, and IHC analysis indicated the synergistic effect between SPT6 and SND1 on the expression of hTERT and stemness marker (Fig. 6E,F). Altogether, these findings demonstrated that SND1 functionalizes as an indispensable co‐factor of SPT6 to regulate the expression of hTERT and tumor growth in colon cancer progression.

### 3.7 SPT6, SND1, and hTERT are all highly expressed in CRC tissues and positively correlated with each other

**Fig. 7:**
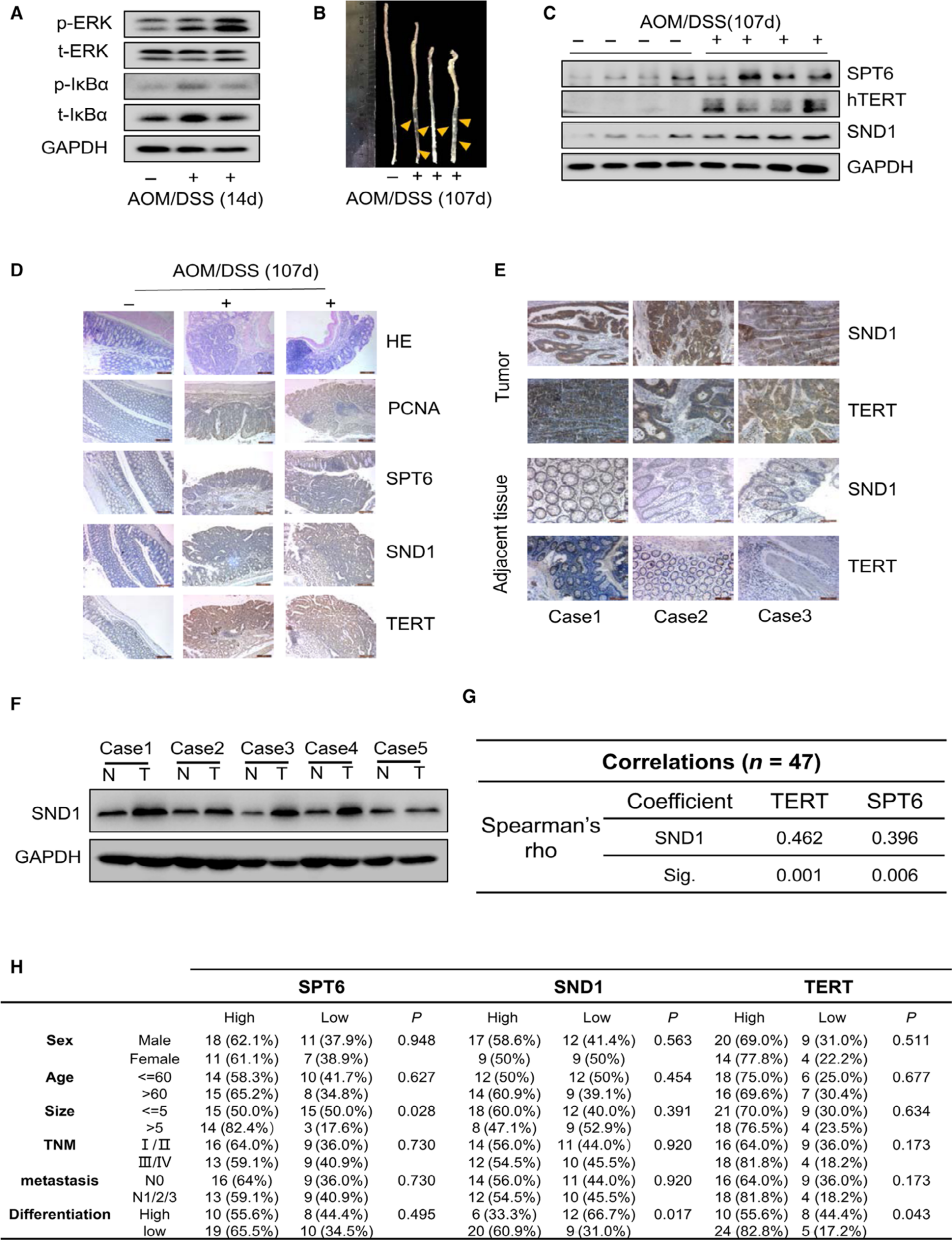
SPT6, SND1 and hTERT are all highly expressed in CRC tissues and positively correlated with each other. (A) Western blot assay of the inflammation‐associated factors in the colon tissues of mice treated by AOM/DSS for 14 days. (B) The representative photographs of mouse colon at the end of AOM/DSS treatment. (C) Western blot assay on the expression of SND1, SPT6, and hTERT in the mouse colon tissues at the end of AOM/DSS treatment. (D) The representative HE and IHC staining for PCNA, SPT6, SND1, and hTERT in mouse colon tissues at the end of AOM/DSS treatment. Scale bars represent 200 μm. (E) IHC staining for SND1 and hTERT in the tumor tissues and the adjacent nontumor tissues from patients with CRC. Scale bars represent 100 μm. (F) Western blot assay on the expression of SND1 in the tumor tissues and the adjacent nontumor tissues from patients with CRC. (G) The expression correlation between SND1 and hTERT or SPT6 according to the quantification assay of IHC staining. (H) The relationship analysis between SPT6, SND1, or hTERT expression and the clinical–pathological parameters in colon cancer patients.

Colitis is known to provide the basis for the development of colorectal tumorigenesis. Patients with chronic inflammatory bowel disease are at risk of developing CRC [46, 47]. Hence, the mouse model with Colon cancer in situ originating from inflammation plays a key role in exploring the underlying molecular pathogenesis of tumorigenesis and development. We established such a model by applying AOM/DSS induction (Fig. S6A) to further investigate the synergy effect of SPT6 and SND1 on the expression of hTERT during colon cancer progression. On the 14th day after treatment initiation, we collected the colon tissues and detected the expression of inflammatory markers, and found the levels of p‐ERK and p‐IκBα was significantly increased, indicating the occurrence of inflammatory progression (Fig. 7A, Fig. S6B). At the end of treatment, we collect the colon tissues of the mice and observed obvious colon contractions and nodules in the treated group compared with the control group (Fig. 7B). Furthermore, through WB and IHC analysis, we observed significantly increased expression of SPT6, SND1, and hTERT in the formed colon nodules, but not in the normal tissues (Fig. 7C,D). More notably, the expression between SPT6 and SND1 or SPT6 and hTERT in the formed tumors showed significant positive correlations (Table 2, Fig. S6C,D) Here, we also evaluated the expression of SND1 and its expression correlation with hTERT in the clinical samples, which were also used to examine the expression of SPT6 before. WB and IHC analysis showed the high expression of SND1 and hTERT in tumor tissues compared to the adjacent normal tissues (Fig. 7E–G). Moreover, consistent with the results from the CRC mouse model, the quantitative analysis of expression in IHC slides showed a positive correlation between SND1 and hTERT or SND1 and SPT6, confirming again the potential regulation of SPT6 and SND1 on hTERT. Then, the gene expression level was divided into two groups (high and low). The relationship between the expression of SPT6, SND1, or hTERT and the clinicopathological characteristics of patients was analyzed. As shown in Fig. 7H, the expression of SPT6 was relevant to the size of tumors, and the expression of SND1 and hTERT was relevant to the differentiation of tumors. Thus, these *in vivo* data from CRC tissues collectively demonstrated the possibility that SPT6 cooperates with SND1 to regulate the expression of hTERT and to be further involved in the development of colon cancer.

**Table 2:** Score of IHC staining for the expression of SPT6, SND1, and hTERT based on tissue samples from mouse with colon cancer. N, control group, T, treatment group.

Sample	Expression of SPT6	Expression of SND1	Expression of hTERT
N_1	1	3	3
N_2	3	2	1
N_3	1	2	2
N_4	2	3	4
N_5	3	1	3
T_1	6	8	5
T_2	7	5	8
T_3	8	6	6
T_4	5	6	5
T_5	5	8	6
T_6	7	7	9
T_7	7	6	5
T_8	6	6	10
T_9	7	8	9
T_10	9	9	8

## 4 Discussion

In this study, we describe a novel mechanism by which SPT6 associates with SND1 to promote CRC *via* targeting hTERT. SPT6 plays a key role in transcription initiation as a histone chaperon to modify chromatin structure *via* associating with RNAPⅡ, histones, and Iws1 [24, 48]. Nevertheless, the precise downstream target genes controlled by SPT6 in gene activation remain poorly defined, albeit the latest report of IFN‐stimulated genes [27]. Hence, to our knowledge, this might be the first documentation reporting that SPT6 controls the expression of hTERT by functionalizing as its specific transcriptional regulation factor in colon cancer cells and further participates in the regulation of CRC progression.

As a critical factor that promotes cell immortalization, the ubiquity of hTERT reactivation in cancer makes it an attractive target in cancer therapy with great clinical potential [8, 49]. The tumor cell growth arrest, loss of tumorigenic properties, and the increased efficiency of drug‐resistant cancer cells elimination upon hTERT expression inhibition have been broadly exhibited *in vitro* and *in vivo*, and even in preclinical or clinical trials [50, 51, 52], necessitating the better understanding of hTERT transcription initiation and function exertion for the presentation of alternative strategy or combinational strategy to more effectively target hTERT. In line with this emergency requirement, our study proved that the transcription of hTERT in CRC is strictly controlled by SPT6 and SPT6/hTERT signaling axis serves as a potential therapeutic target in CRC evidenced by the direct binding of SPT6 at the promoter region of hTERT resulting in the transcription initiation and translation changes of hTERT and subsequent influence on the function of colon cancer cells *in vitro* and *in vivo*.

SPT6 silencing not only led to the proliferation inhibition, apoptosis induction, the decrease of stem‐like straits, and the improvements of chemotherapeutic sensitivity in CRC cells *in vitro* but also caused growth arrest and metastasis delay and reduction in the mouse model of CRC cells. Of note, consistent with what we detect that hTERT expression was strictly controlled by SPT6, the ectopic overexpression of hTERT effectively reversed the above functional changes mediated by SPT6 silencing ex vivo, illustrating again the key role of hTERT in CRC tumorigenesis and development, and also implying the potential regulation of hTERT by SPT6 in CRC cells. Most likely, hTERT functionalizes as the pivotal, perhaps not the only, downstream target of SPT6, to mediate its action in driving CRC progression. In other words, very possibly, without hTERT, SPT6 could not perform its tumor‐promoting role in CRC development. Considering that the loss of hTERT activity is an important tumor suppressor strategy, our findings thereby garner the significant interest of the dual blockade of SPT6 and hTERT as a novel strategy to combat cancer, especially CRC.

Given the complexity of the transcription mechanism in the tumor [39, 40], the broad interaction of SPT6 with other proteins in the maintenance of chromatin structure, and the possibility that SPT6 binds to hTERT promoter indirectly, we proposed the existence of the synergistic factors for SPT6 in initiating hTERT transcription and further identified SND1 as such synergy factor targeting hTERT through the binding at the promoter of hTERT. As a component of the RNA‐induced silencing complex, SND1 is reported to be highly expressed in human colon cancers and implicated in early‐stage colon carcinogenesis by participating in the posttranscriptional modifications of APC and ß‐catenin 50. Here, consistently, we also found the upregulation of SND1 in CRC tissues compared to the paired noncancerous tissues and its oncogenic function in CRC progression. But on the contrary to the previous investigations, our study revealed the contribution of SND1 to the transcriptional regulation of key genes involved in cancer development, focusing on hTERT. It was proved to be recruited by SPT6 to anchor at hTERT promoter region, synergize with SPT6 to co‐regulate hTERT expression, and further influence the function of CRC cells *in vitro* and *in vivo*. These data enrich our knowledge about the precise function and the corresponding molecular mechanisms of SND1 in CRC, most possibly different at different stages of tumor development. Meanwhile, it is not exclusive that, besides SND1, some other transcriptional factors are similarly recruited by SPT6 to form a complex with SPT6 and SND1 at hTERT promoter and to co‐mediate gene transcription. Within this complex, which protein is responsible for binding to hTERT promoter directly, and which ones functionalize as cofactors? All these possibilities and hypotheses deserve to be deeply explored in our future study.

Clinically, based on the statistical analysis in a cohort of CRC cases [52], the elevated expression of SND1, SPT6, and hTERT was respectively observed, and the expression of SND1 and hTERT was found to be significantly associated with the differentiation degree of tumors. Besides, more significantly, the positive correlation between each other among the expression of these three factors was further revealed, indirectly showing the synergy of SPT6 and SND1 in initiating hTERT transcription in CRC progression. In line with this finding, AOM/DSS model in mice, a powerful tool to investigate the mechanisms of colon carcinogenesis and development [53, 54], also proved the positive correlation among the expression of hTERT, SPT6, and SND1, implying again the potential regulation of hTERT by SPT6 and SND1 in CRC. Thus, considering the role of hTERT itself as a central driving force of CRC, our current explorations at least suggest that the combinational inhibition simultaneously targeting SPT6, SND1, and hTERT might provide an alternative and novel strategy in CRC treatment. Of course, more explorations in a larger cohort of patients with CRC should be performed in the future study to further confirm such regulatory relationship and the oncogenic role of SPT6 *via* synergizing with SND1.

Concerning the role of SND1 in SPT6/hTERT signaling, our results not only demonstrated the oncogenic function of SND1 itself in CRC survival but also uncovered its indispensability in the regulation of hTERT expression and tumor growth mediated by SPT6, as evidenced by the reverse of the upregulation of hTERT expression, cell viability, stem‐like properties, and chemotherapeutic resistance caused by SPT6 overexpression upon SND1 knockdown. In summary, our study revealed that SPT6 synergized with SND1 to upregulate the transcription and translation of hTERT and in turn played a critical role in the promotion of CRC progression. All the findings provide the mechanistic insights into the expression regulation of hTERT in CRC initiation and development, demonstrate that disrupting the SPT6/SND1 transcriptional activation axis might be a compelling strategy to target hTERT‐dependent cancer, especially CRC, and also prompt the application of multiple inhibitors, respectively, against SPT6, SND1, and hTERT for optimal anticancer effects.

## 5 Conclusions

In conclusion, this study demonstrated that SPT6 promoted CRC progression by transcriptionally activating hTERT, and such transcriptional activation needed the involvement of SND1, which functionalizes as the synergistic transcriptional factor to co‐anchor at the hTERT promoter region (−234 to −144) with SPT6 to co‐regulate hTERT expression and the sequential CRC progression. All the data not only provided the molecular mechanism insights into the expression regulation of hTERT in CRC initiation and development, but also put forward the possibility that inhibiting SPT6‐SND1‐hTERT axis simultaneously might create a therapeutic vulnerability in CRC.

## Conflict of interest

The authors declare no conflict of interest.

## Author contributions

This work was carried out in collaboration between all authors. CD and WG defined the research theme, designed the experimental approach, and revised the manuscript critically. CD, PG, WY, YS, YL, WY, JH, SH, MC, YZ, RW, WL, JP, CH, and XL carried out the experiments. CD, WY, PG, YS, WY, JH, WD, GL and WG analyzed the data and interpreted the results. CD, GL, and WG wrote the manuscript. All authors read and approved the final manuscript.

## Supporting information


**Fig. S1.** SPT6 transcriptionally regulates hTERT in colon cancer cells.

**Fig. S2.** SPT6 knockdown induces apoptosis, stemness arrest, and chemotherapeutic sensitivity improvement in colon cancer cells *in vitro*.

**Fig. S3.** SND1 promotes colon cancer cell proliferation and stemness *via* transcriptionally regulates hTERT.

**Fig. S4.** SND1 recruits and interacts with SPT6 to co‐regulate hTERT expression and colon cancer cell survival *in vitro*.

**Fig. S5.** SND1 synergized with SPT6 to co‐regulate the sensitivity of colon cancer cells to chemotherapeutics.

**Fig. S6.** SPT6, SND1 and hTERT are all highly expressed in CRC tissues and positively correlated with each other.
