# Correction to “SPT6 recruits SND1 to co‐activate human telomerase reverse transcriptase to promote colon cancer progression”

**DOI:** 10.1002/1878-0261.13823

**Published:** 2025-03-07

Diao C, Guo P, Yang W, Sun Y, Liao Y, Yan Y, Zhao A, Cai X, Hao J, Hu S, Yu W, Chen M, Wang R, Li W, Zuo Y, Pan J, Hua C, Lu X, Fan W, Zheng Z, Deng W, Luo G, Guo W. SPT6 recruits SND1 to co‐activate human telomerase reverse transcriptase to promote colon cancer progression. *Mol Oncol*. 2021;15:1180–1202. https://doi.org/10.1002/1878-0261.12878.

The article by Diao et al. contained inadvertent duplications between two western blot images presented in Fig. 3C and 3E, and between two IHC staining images presented in Fig. 4G.

The authors have corrected this by providing the original raw data for all experimental replicates, and the revised figures are included here. All authors agree to this corrigendum and confirm that changes do not affect the conclusions of the article.

The corrected figures are reproduced below.

**Figure:**
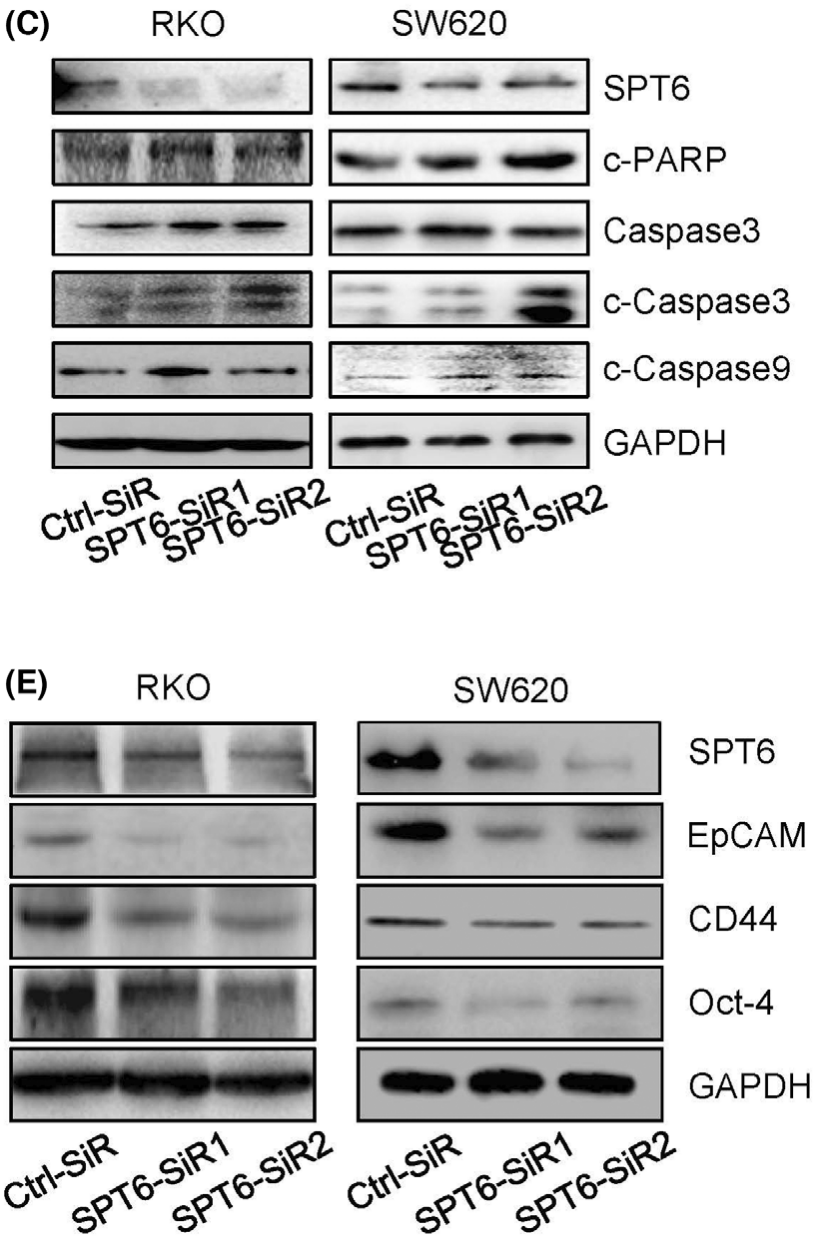
Figure 3

**Figure:**
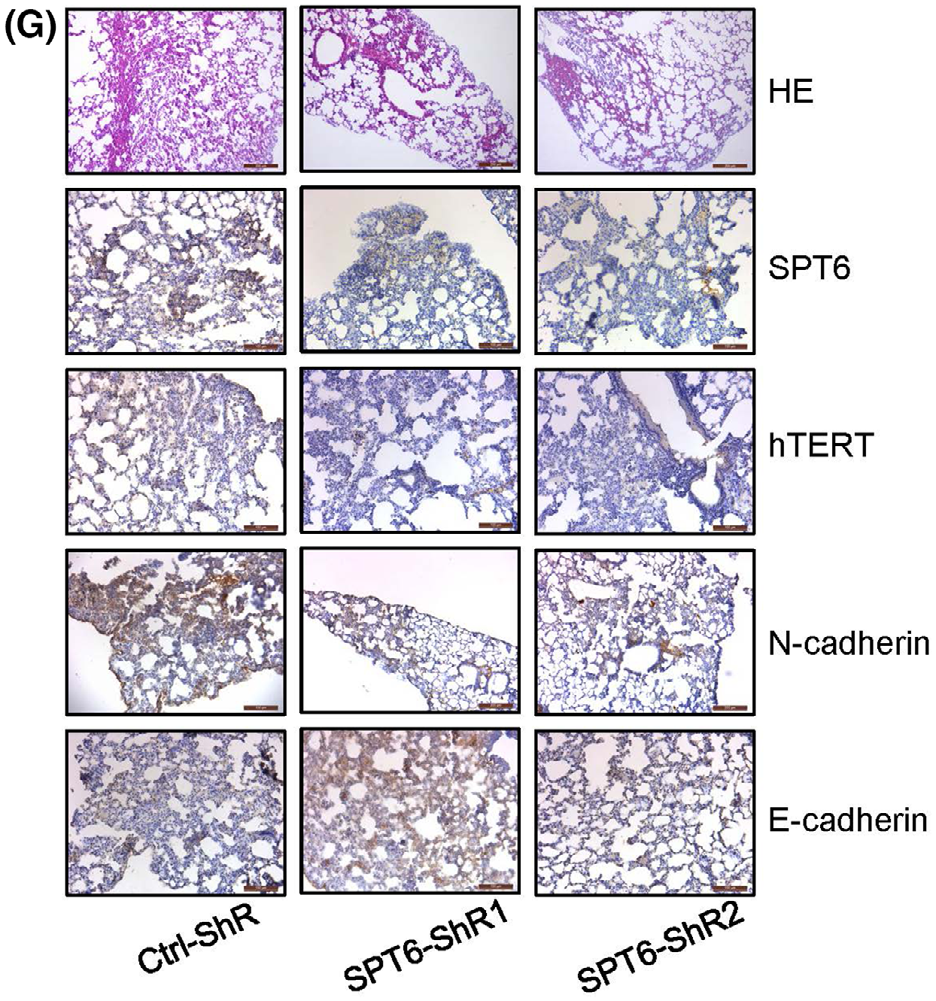
Figure 4

The authors apologize for any inconvenience caused.

