# Supplementary figures and images for: SPT6 recruits SND1 to co‐activate human telomerase reverse transcriptase to promote colon cancer progression
Source: Mol Oncol. 2021 Jan 12;15(4):1180–202. doi: 10.1002/1878-0261.12878 (PMC8024721; doi:10.1002/1878-0261.12878)

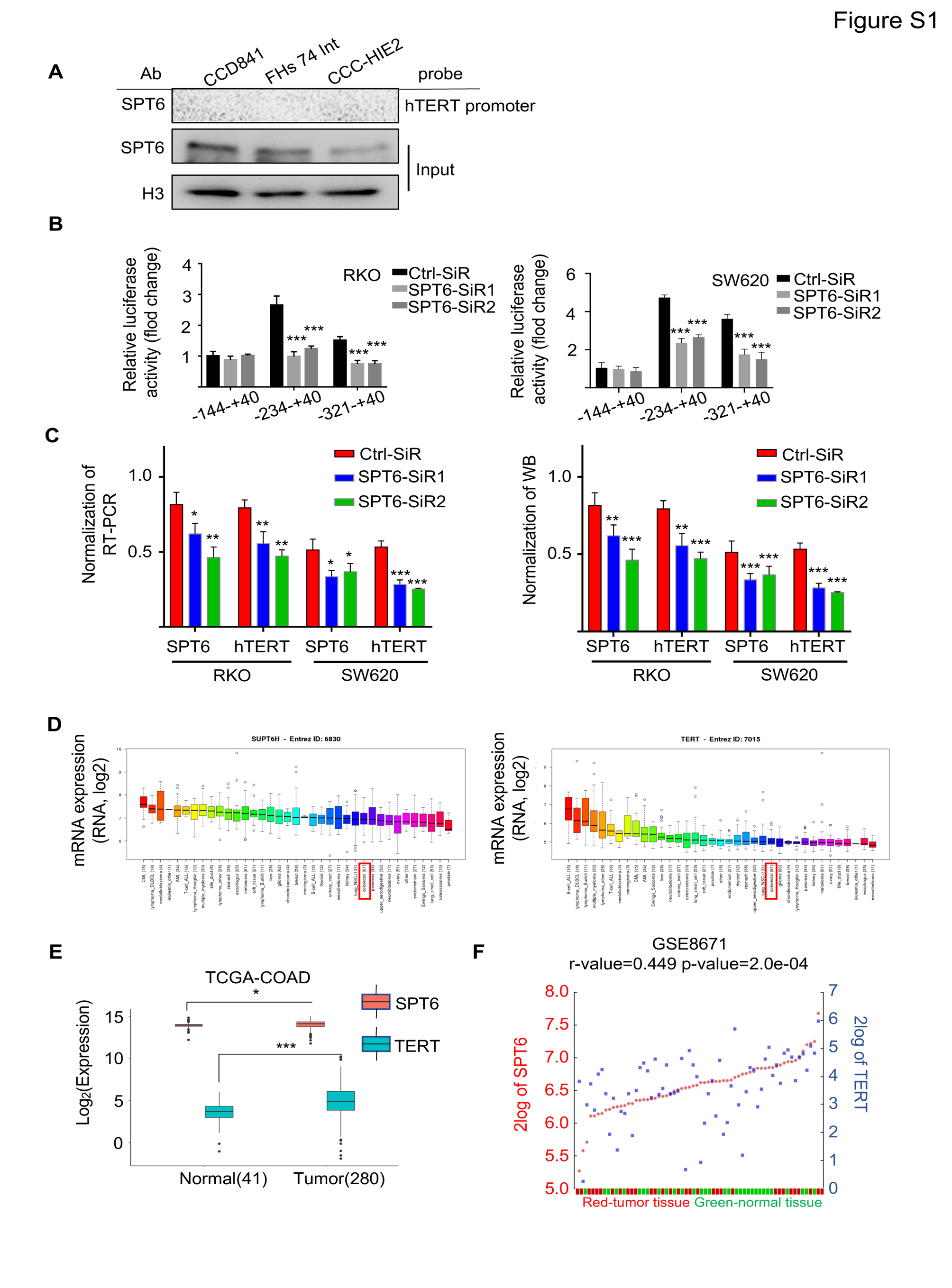

Supplement: Supplementary file 1 — Fig. S1. SPT6 transcriptionally regulates hTERT in colon cancer cells. [file MOL2-15-1180-s001.tif]

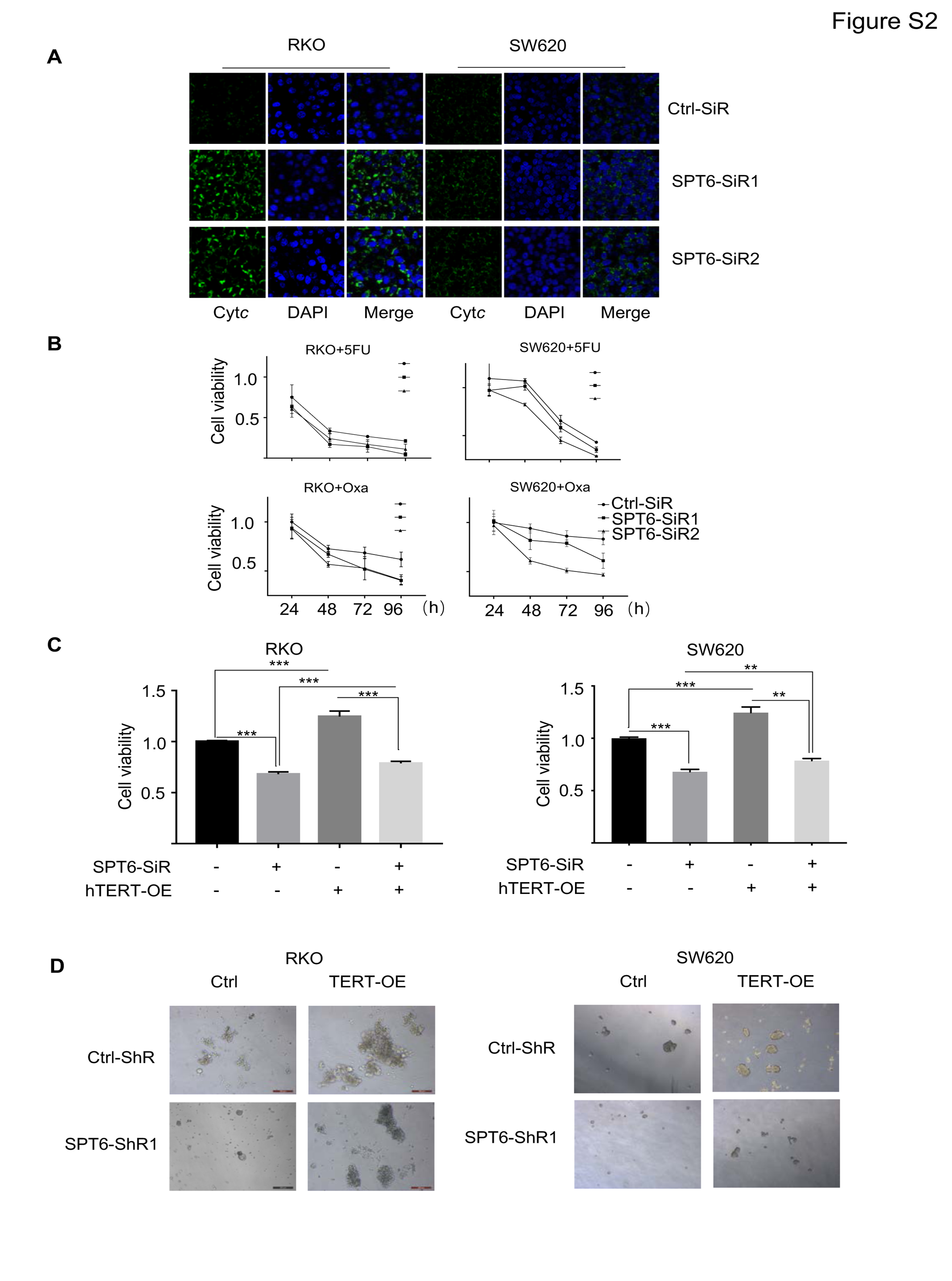

Supplement: Supplementary file 2 — Fig. S2. SPT6 knockdown induces apoptosis, stemness arrest, and chemotherapeutic sensitivity improvement in colon cancer cells in vitro. [file MOL2-15-1180-s003.tif]

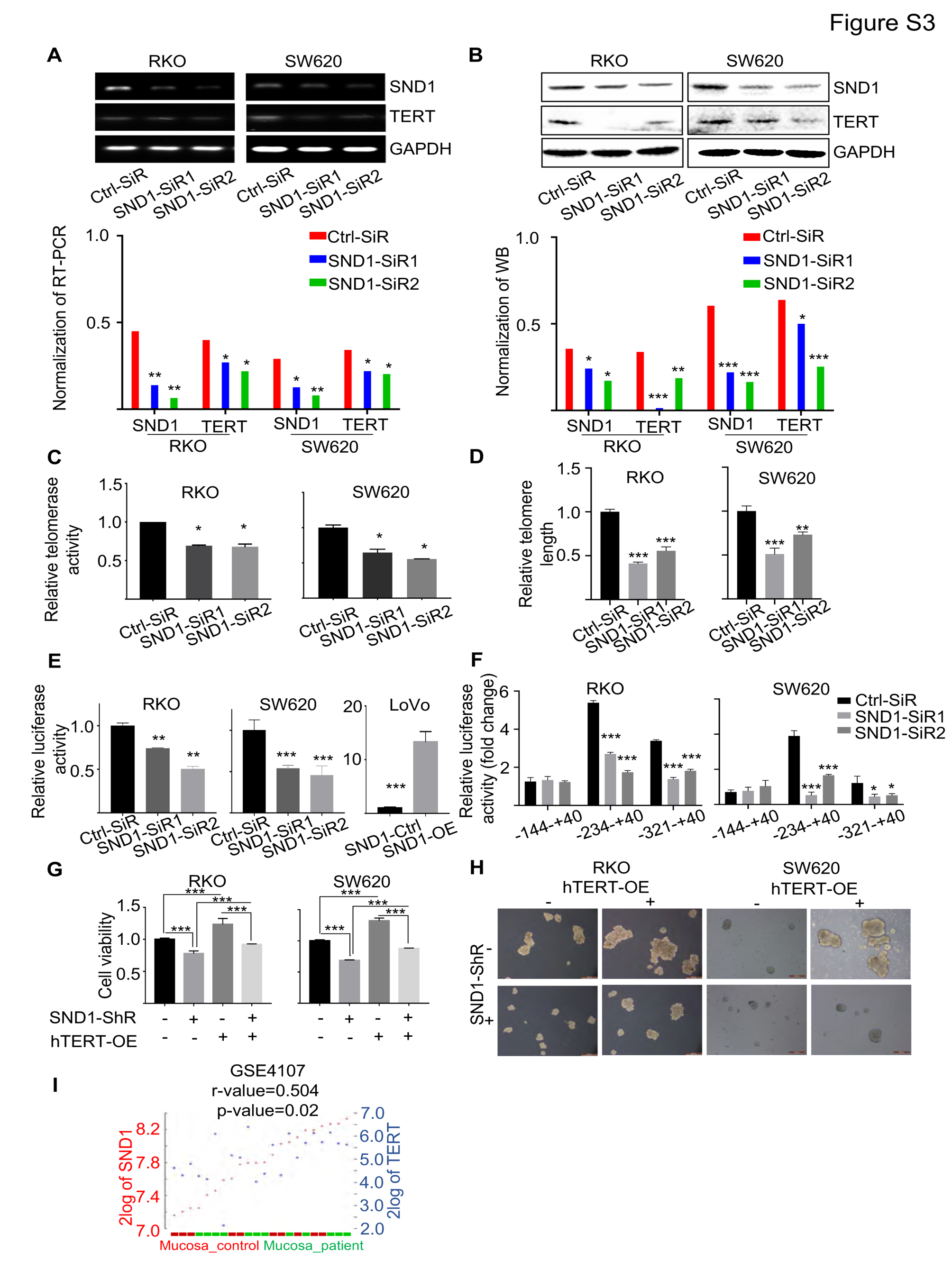

Supplement: Supplementary file 3 — Fig. S3. SND1 promotes colon cancer cell proliferation and stemness via transcriptionally regulates hTERT. [file MOL2-15-1180-s002.tif]

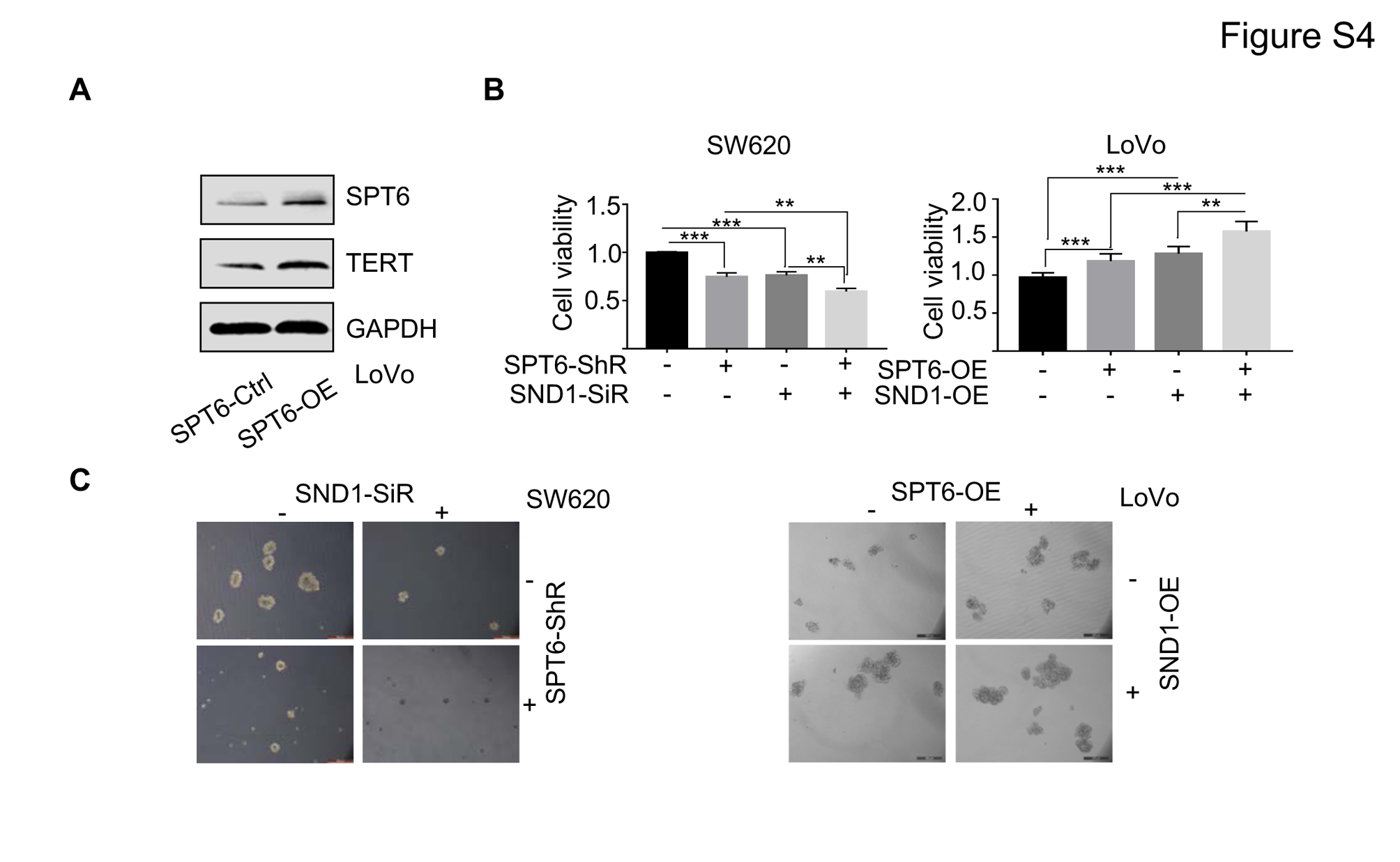

Supplement: Supplementary file 4 — Fig. S4. SND1 recruits and interacts with SPT6 to co‐regulate hTERT expression and colon cancer cell survival in vitro. [file MOL2-15-1180-s004.tif]

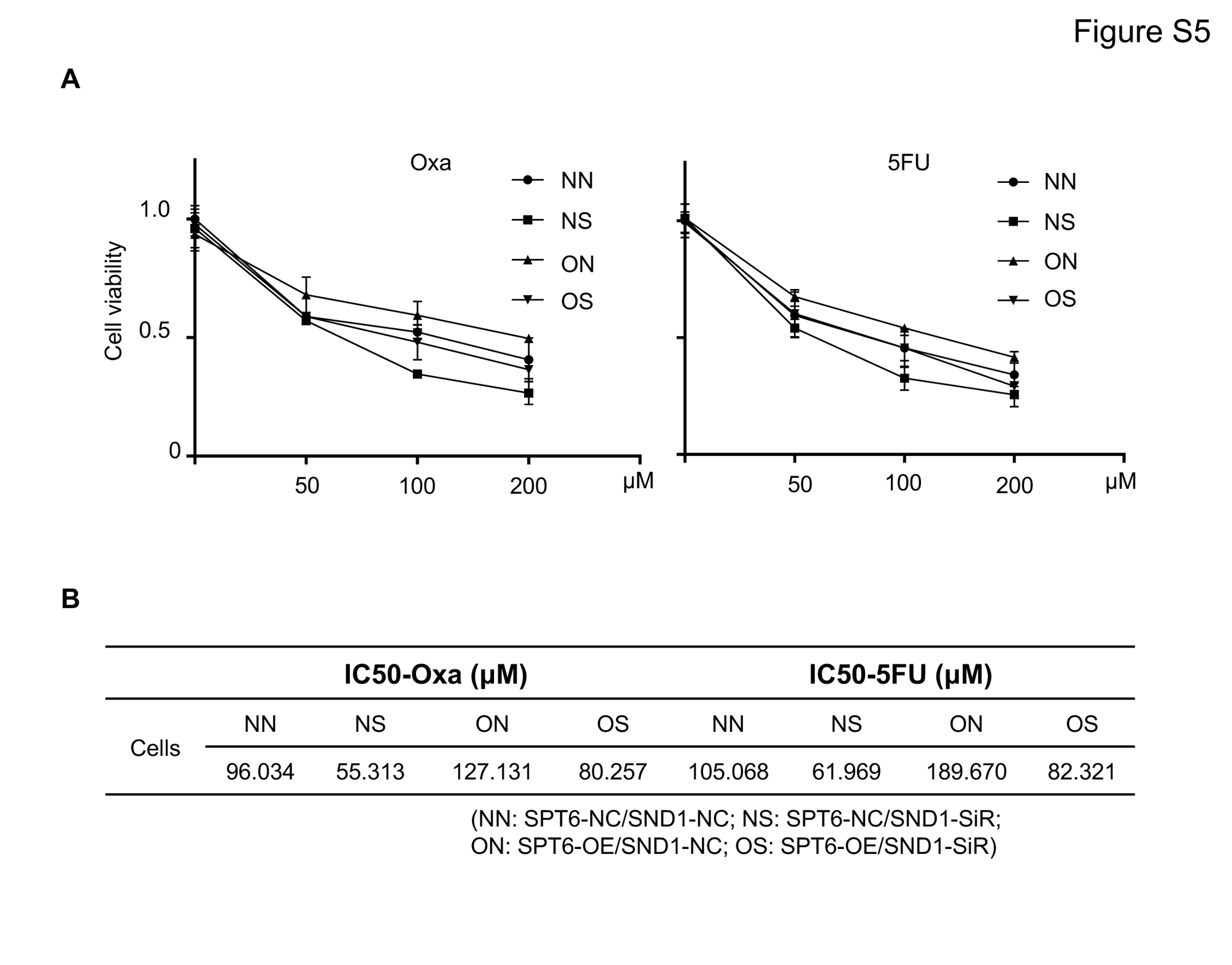

Supplement: Supplementary file 5 — Fig. S5. SND1 synergized with SPT6 to co‐regulate the sensitivity of colon cancer cells to chemotherapeutics. [file MOL2-15-1180-s006.tif]

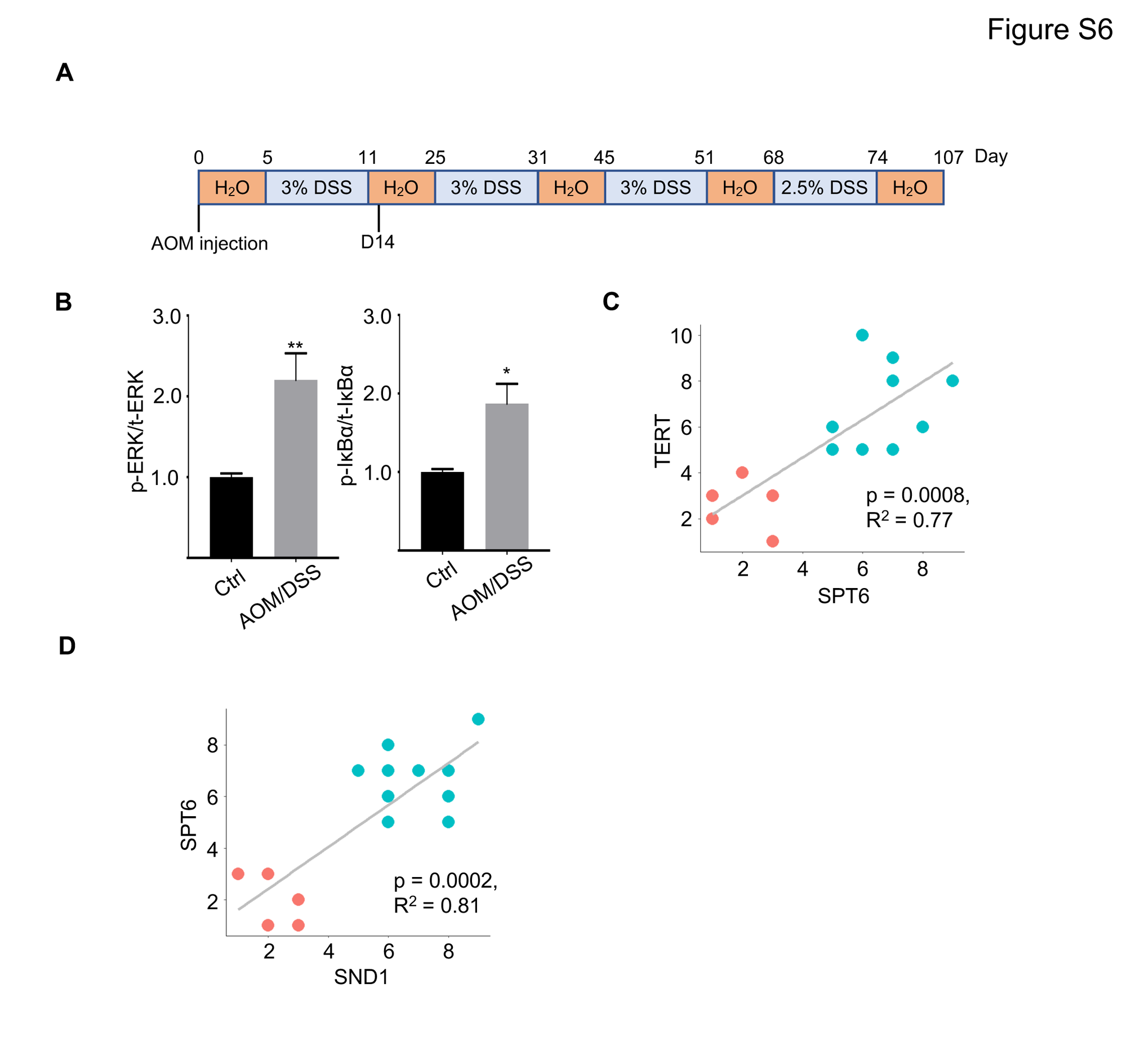

Supplement: Supplementary file 6 — Fig. S6. SPT6, SND1 and hTERT are all highly expressed in CRC tissues and positively correlated with each other. [file MOL2-15-1180-s005.tif]
